# Estrogen-related genes for thyroid cancer prognosis, immune infiltration, staging, and drug sensitivity

**DOI:** 10.1186/s12885-023-11556-0

**Published:** 2023-10-31

**Authors:** Leiying Zhang, Man Zhou, Xiaoni Gao, Yang Xie, Junqi Xiao, Tao Liu, Xiangtai Zeng

**Affiliations:** 1 Suzhou Medical College of Soochow University, Suzhou, China; 2 The First Affiliated Hospital of Gannan Medical University, Ganzhou, China; 3 College of Pharmacy, Gannan Medical University, Ganzhou, China; 4 Institute of Thyroid Diseases, Gannan Medical University, Ganzhou, China; 5 Ganzhou Key Laboratory of Thyroid Tumor, Ganzhou, China; 6 Beijing Business University, Beijing, China

**Keywords:** Estrogen-related genes, Estrogen-related differential expressed genes, Progression-free interval, Thyroid cancer, Immune infiltration, Clinical relevance, Drug sensitivity

## Abstract

**Background:**

Thyroid cancer (THCA) has become increasingly common in recent decades, and women are three to four times more likely to develop it than men. Evidence shows that estrogen has a significant impact on THCA proliferation and growth. Nevertheless, the effects of estrogen-related genes (ERGs) on THCA stages, immunological infiltration, and treatment susceptibility have not been well explored.

**Methods:**

Clinicopathological and transcriptome data of patients with THCA from the Gene Expression Omnibus (GEO) and The Cancer Genome Atlas (TCGA) were cleaned before consensus clustering. Differential expression analysis was performed on the genes expressed between THCA and paraneoplastic tissues in TCGA, and Wayne analysis was performed on the ERGs obtained from the Gene Set Enrichment Analysis MsigDB and differentially expressed genes (DEGs). Univariate Cox and least absolute shrinkage and selection operator (LASSO) analyses were used to identify the set of estrogen-related differentially expressed genes (ERDEGs) associated with progression-free intervals (PFI) and to establish a prediction model. Receiver operating characteristic curves were plotted to calculate the risk scores and PFI status to validate the predictive effect of the model. Enrichment analyses and immune infiltration analyses were performed to analyze DEGs between the high- and low-risk groups, and a nomogram plot was used in the risk model to predict the PFI of THCA.

**Results:**

The expression of 120 ERDEGs differed significantly between the two groups (*P <* 0.05). Five (CD24, CAV1, TACC1, TIPARP, and HSD17B10) of the eight ERDEGs identified using univariate Cox and LASSO regression were validated via RT-qPCR and immunohistochemistry analysis of clinical tissue samples and were used for clinical staging and drug sensitivity analysis. Risk-DEGs were shown to be associated with immune modulation and tumor immune evasion, as well as defense systems, signal transduction, the tumor microenvironment, and immunoregulation. In 19 of the 28 immune cells, infiltration levels differed between the high- and low-risk groups. High-risk patients in the immunotherapy dataset had considerably shorter survival times than low-risk patients.

**Conclusion:**

We identified and confirmed eight ERDEGs using a systematic analysis and screened sensitive drugs for ERDEGs. These results provide molecular evidence for the involvement of ERGs in controlling the immunological microenvironment and treatment response in THCA.

**Supplementary Information:**

The online version contains supplementary material available at 10.1186/s12885-023-11556-0.

## Introduction

Thyroid cancer (THCA) is the most common endocrine tumor, and its rate of occurrence has steadily increased worldwide over the last 30 years [1]. Between 2000 and 2016, the number of cases of THCA in China increased by 20 times and was most noticeable among women. According to China’s national cancer statistics, the rate of female THCA increased from the seventh most common cancer in 2012 to the third most common cancer in 2016. In a statistical study of Chinese tumor registries performed in 2020, THCA had the second highest incidence rate among Chinese patients with cancer aged 15–44 years, after breast cancer in women and liver cancer in men [2]. Although the mortality rate of thyroid cancer (THCA) is relatively low, the number of women diagnosed with THCA continues to increase. This trend not only adds to the national medical and family burden but also raises concerns about the health and fertility of women when they reach childbearing age. The expression of estrogen receptor in patients with thyroid cancer is reportedly higher than that in normal thyroid tissue [3], and thyroid hormone receptor can mediate its effect on mitochondrial processes by increasing the expression of the gene encoding estrogen receptor [4]. This indicates that estrogen and related genes play an important role in thyroid cancer, but the specific mechanism needs further exploration. Therefore, studying how estrogen and other female hormones affect the initiation, progression, and outcomes of THCA is crucial. This study aimed to use a database and experimental validation to better understand the role of estrogen-related genes (ERGs) in THCA.

Correlation analysis was used to identify estrogen-related differentially expressed genes (ERDEGs). Furthermore, examining the links between risk scores and molecular functions, pathways, consequences, immunological infiltration, and immunotherapy was a major focus. In addition, the associations between ERDEGs, clinicopathology, and drug sensitivity were investigated. Finally, we used RT-qPCR and immunohistochemistry (IHC) labeling to establish ERDEGs in clinical THCA and paracancerous tissue samples.

## Materials and methods

### Data sources and pre-processing

We obtained the GSE33630 dataset on THCA from the Gene Expression Omnibus (GEO) database using the GEOquery package [5]. The GSE33630 dataset [6] included 105 samples: 11 from undifferentiated THCA, 49 from papillary thyroid cancer (PTC), and 45 from healthy individuals. The data platform used was GPL570. In total, 60 THCA samples and 45 normal samples were used to generate normalized data using the Limma package [7]. A total of 553 normal individuals and patients with tumor (TCGA-THCA, *n =* 553 cases) obtained from The Cancer Genome Atlas (TCGA) using the Biolinks package were included in the THCA RNA-Seq dataset [8]. To acquire information such as sex, survival status, follow-up period, and disease stage from TCGA-THCA-matched patients, the data type counts and fragments per kilobase of exon model per million mapped fragments were chosen and converted to transcripts per kilobase of exon model per million mapped reads (TPM) format.

### Differential expression analysis

From TCGA, we obtained a normal (01A) and tumor group (11A) of THCA. Differential analysis of genes (count values) in different groups was performed using the R package Deseq2 [9]. A threshold of *P <* 0.05 was set for differentially expressed genes (DEGs), where log2FC > 0 and log2FC < 0 denoted differential genes upregulated and downregulated in the disease group, respectively. ERGs were obtained using Gene Set Enrichment Analysis (GSEA) MSigDB (www.gsea-msigdb.org) and intersected with DEGs to obtain ERDEGs. In the follow-up analysis, patients in the tumor group were divided into high- and low-risk groups according to the median risk score of the patients in the model. Differential analysis of genes (count values) in the different groups was performed using the R package Deseq2 [9]. A threshold of *P <* 0.05 was set for DEGs, where log2FC > 1 and log2FC < 1 denoted differential gene upregulated and downregulated in the high-risk group, respectively.

### Risk model construction

Patients in TCGA-THCA were split into training and validation sets based on a 7:3 ratio, and univariate Cox regression analysis was used to examine how different factors affected the progression-free interval (PFI). Variables with a *P <* 0.1 were associated with PFI and included in the follow-up analysis. Least absolute shrinkage and selection operator (LASSO) regression is a machine learning approach commonly used to construct diagnostic models and uses regularization to address the occurrence of overfitting during curve fitting and improve the accuracy of the model. We used the glmnet package [10] to further screen for ERDEGs associated with PFI as key genes with the following parameters set: seed (8), family = "cox".

### Immunotherapy analysis

Datasets relevant to immunotherapy were gathered using the IMvigor 210 CoreBiology package [11]. Patients undergoing immunotherapy had their risk score determined using LASSO–Cox regression coefficients. Treatment outcomes were compared between high- and low-risk patient groups by classifying patients into groups based on their median scores.

### Enrichment analysis 

Gene ontology (GO) analysis is often used for large-scale functional enrichment studies examining biological processes (BP), molecular functions (MF), and cellular components (CC). Many researchers use the Kyoto Encyclopedia of Genes and Genomes (KEGG), a database of genomes, biological processes, diseases, and treatments. Using the clusterProfiler R package [12] to perform GO annotation and KEGG pathway enrichment for DEGs, a *p*-value of 0.05 was considered statistically significant. Both the GO annotation study of DEGs and the KEGG pathway enrichment analysis employed a significance level of *P <* 0.05. Gene set enrichment analysis (GSEA), a computational method commonly used to estimate changes in pathways and BP activity in samples of gene expression datasets, was applied to gene expression profile data from the high- and low-risk groups of TCGA-THCA patients to investigate the differences in BP between different subgroups. Using GSEA, based on the MSigDB database, we found that the gene set "c2.all.v7.2. symbols.gmt" was highly enriched (*P <* 0.05).

### Immune infiltration (Single-sample gene set enrichment analysis, ssGSEA)

We used the GSVA package [13] to perform ssGSEA on gene expression data from patients with THCA and estimate the composition and abundance of 28 types of immune cells. We then compared the immune cell differences between high- and low-risk groups, as well as the key genes associated with immune cells.

### Nomogram

Nomogram plots, also called column plots, were constructed based on the results of the multivariate analyses. In these analyses, multiple predictors were combined and assigned based on certain proportions to show how variables related to each other in a graphical format predict the outcome. We used multivariate Cox regression to determine how often THCA worsened based on the risk score, sex, and pathological stage. We plotted the nomogram and used calibration curves to determine the accuracy of the model.

### GEO validation of ERDEG and correlation analysis with stage

We conducted a validation of gene expression patterns linked to PFI using the GEO dataset. Subsequently, we identified key genes with statistically significant expression differences and consistent trends. These key genes, along with their associated risk scores, were further analyzed in relation to pathological, T, N, and M stages. Baseline information is presented in Table 1.

**Table1:** TCGA-THCA baseline information table

	**Overall**
n	496
PFI.time (mean (SD))	3.11 (2.63)
Stage (%)	
I	279 (56.5)
II	52 (10.5)
III	110 (22.3)
IV	53 (10.7)
Tstage (%)	
T1	141 (28.5)
T2	162 (32.8)
T3	169 (34.2)
T4	22 (4.5)

### Drug sensitivity analysis

The CellMiner database (https://discover.nci.nih.gov/cellminer/) was used to query mRNA expression patterns and drug activity in NCI-60 cells. CellMiner is a web-based resource that provides genomic and pharmacological data based on NCI-60 transcripts and drug response data. The National Cancer Institute has collected transcription and medication response data. The CellMiner website provides access to the transcript expression levels of 22,379 genes, 360 miRNAs, and the pharmacological reactions of 20,503 chemicals. Using Pearson’s correlation coefficient, we determined the relationship between gene expression and chemical sensitivity.

The changes in the cancer genome strongly affect the clinical response to treatment, and in many cases are effective biomarkers for drug response. The Genomics of Drug Sensitivity in Cancer (GDSC) database (www.cancerRxgene.org) is the largest public resource for information on cancer cell drug sensitivity and molecular markers of drug response. The GDSC database can be used to find tumor drug response data and genomic sensitivity markers. We used the pRRophetic algorithm to predict the sensitivity of patients in different clinical variable groups to common anti-cancer drugs or small molecule compounds by calculating IC50 values based on the expression matrix of the TCGA-THCA dataset and displayed the results through group comparison graphs. The cut-off for statistical significance was set at *P <* 0.05.

### Human tissues

The Thyroid Surgery Department of the First Affiliated Hospital of Gannan Medical University in Ganzhou, China, provided 28 pairs of THCA and normal thyroid tissues from the same patients. Rapid freezing in liquid nitrogen was used to preserve all samples. The neighboring tissues were more than 1 cm from the edge of the tumor. Pathologists evaluated the samples and found no visible tumor cells. This study was approved by the respective research ethics boards of the hospitals. All patients provided written informed consent.

### Reverse transcription-qPCR (RT-qPCR)

Total RNA was extracted from the tissues using TRIzol reagent (TransGen Biotech, Beijing, China). Spectrophotometric analysis of the RNA was performed using a Smart Spec Plus device (Bio-Rad, Hercules, CA, USA). The A260/A280 atomic absorption ratio was used to determine purity. Data were analyzed using reverse transcription (RT) reactions. The mRNA reverse transcription procedure was performed using Oligo dT primers. qPCR was conducted using a Prestart Green PCR kit (Transgene Biotech, Beijing, China) and an Applied Biosystems 7300 real-time PCR machine (Applied Biosystems, Foster City, CA, USA). Primers for messenger RNA were as follows: CD24 forward primer: CTCCTACCCACGCAGATTTATTC, and reverse primer: AGAGTGAGACCACGAAGAGAC; CAV1 forward primer: GCGACCCTAAACACCTCAAC and reverse primer: ATGCCGTCAAAACTGTGTGTC; TACC1 forward primer: TCAGCGAATCAGACAAGACAGC and reverse primer: CCGGGTCTCTTCGTATTC; TIPARP forward primer: AGAACGAGTGGTTCCAATCCA and reverse primer: TGGGTGCAAAAGATCAGTCTG; HSD17B10 forward primer: CTGGTGAGATGCCAGAATG and reverse primer: CCAACCTGACCCTCGAAGG.

### Immunohistochemistry (IHC)

Two different tissue pathologists confirmed the diagnosis in each case by examining slides stained with hematoxylin and eosin. Representative formalin fixed and paraffin embedded sections were obtained from each sample for the analysis. Freshly cut slices (5 μm thick) were taken from the tumor blocks buried in each formalin-fixed wax bag. In accordance with a standard scheme, IHC was performed on tissue cuttings buried in wax bags. Immunostained sections were scanned at 200× magnification using a digital scanning system (Tissue FAXS Plus, Vienna, Hungary) and analyzed quantitatively using the ImageJ software.

### Statistical analyses

R software (https://www.r-project.org/, version 4.0.2) was used for all data computations and statistical analyses. Differences between continuous factors in the two groups were examined using the Mann–Whitney U test (Wilcoxon rank-sum test). The Kruskal–Wallis test was used to compare three or more groups based on a constant measure. Receiver operating characteristic (ROC) curves were drawn, and the area under the curve (AUC) was computed in R [14] to evaluate the precision of the risk score predictions. All statistical tests had a significance level of *P* <0.05, and all *p*-values were two-tailed. GraphPad Prism 8's t-test was used to compare RT-qPCR findings from different tissues, and ImageJ was used to evaluate and compare the IHC results. The significance level was set at *P <* 0.05.

## Results

### Technology roadmap

**Fig. 1:**
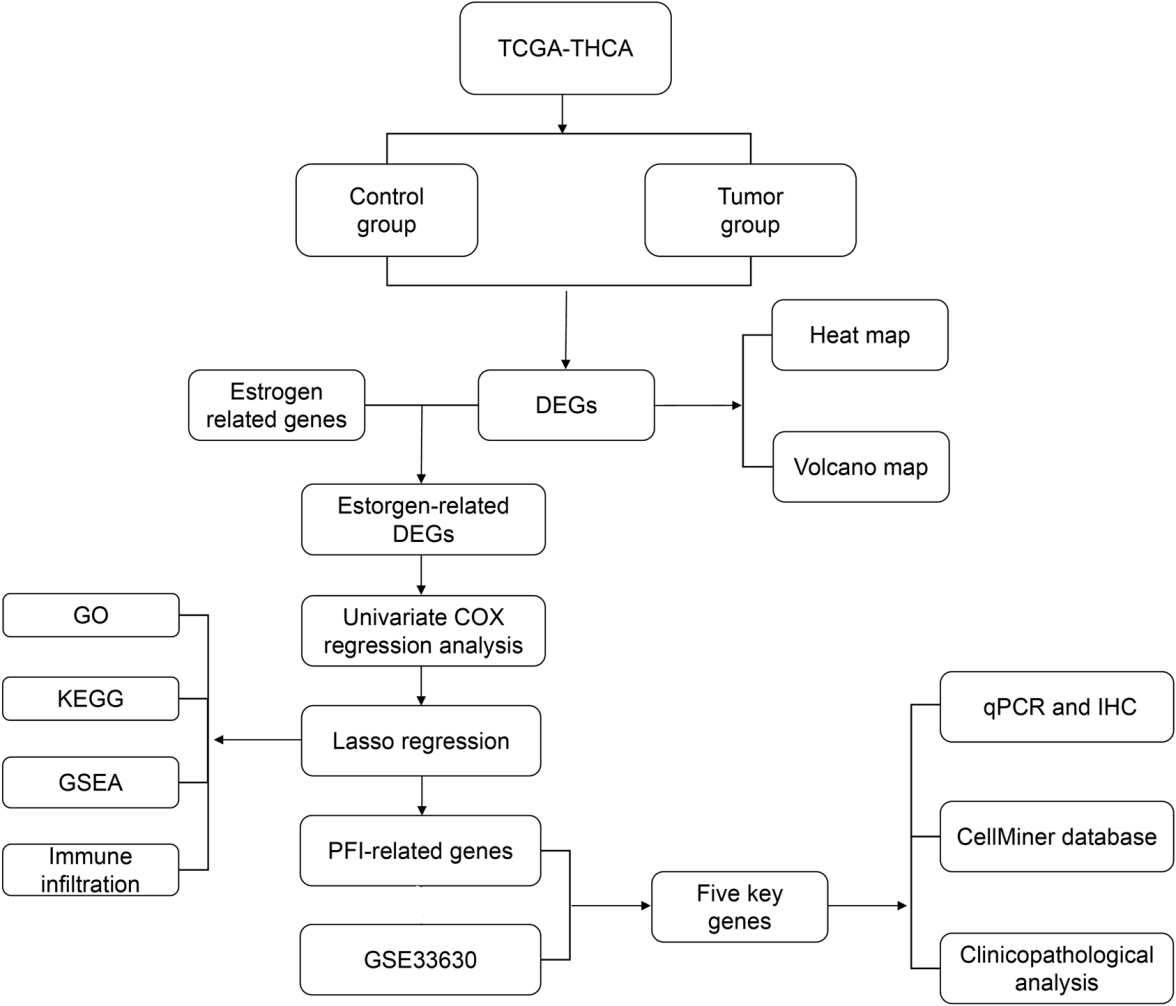
Flow chart of overall project analysis

Figure 1 shows a flowchart of the study. ERGs that showed significant differences between normal and tumor samples in the TCGA-THCA cohort expression analysis (|Log2FC| > 1, FDR < 0.05) were selected. Thereafter, a risk model was built using LASSO regression analysis, and the eight ERDEGs and risk scores were computed. Patients in the TCGA-THCA cohort were divided into high- and low-risk groups according to the median value of their risk ratings and then used for additional GO, KEGG, GSEA, immune infiltration, and immunotherapy research. The ERDEGs were verified using data from the GEO cohort. Finally, THCA and normal tissues were used for in vitro validation.

### ERDEGs in THCA

**Fig. 2:**
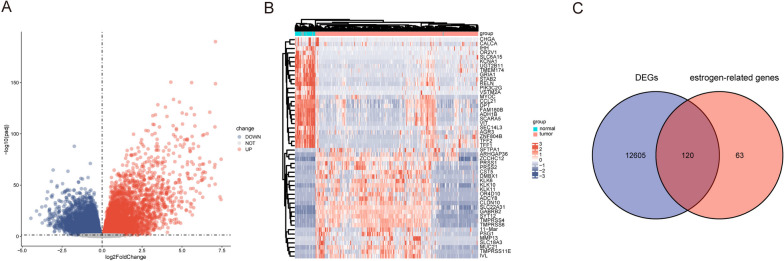
Estrogen-related differentially expressed genes. **A** log2 fold change vs. ˗log10 (adjusted *P*-value); red nodes indicate upregulated and differentially expressed genes, blue nodes indicate downregulated and differentially expressed genes, and grey nodes indicate genes that are not significantly differentially expressed. **B** Patient IDs with associated differentially expressed genes; red represents high gene expression, blue represents low gene expression, pink bars represent tumor tissue, and blue bars represent normal tissue. **C** Blue circles indicate differentially expressed genes in TCGA normal and disease groups, red circles indicate ERGs, and the intersection was taken to obtain ERDEGs. TCGA: The Cancer Genome Atlas; ERGs: estrogen-related genes; ERDEGs: estrogen-related differentially expressed genes

First, we used the DEseq2 program for differential analysis to identify genes whose expression levels were different between tumor and normal tissue samples. A total of 12,725 genes were found to be differentially expressed, with 6,480 upregulated and 6,245 downregulated genes; all DEGs are displayed on a volcano map (Fig. 2A). Heat maps were constructed for the 25 genes with the largest log2FC and 25 genes with the smallest log2FC (Fig. 2B). After crossing 12,725 THCA-related genes with 183 ERGs, 120 ERDEGs were identified (Fig. 2C).

### Prognostic model construction and validation

**Fig. 3:**
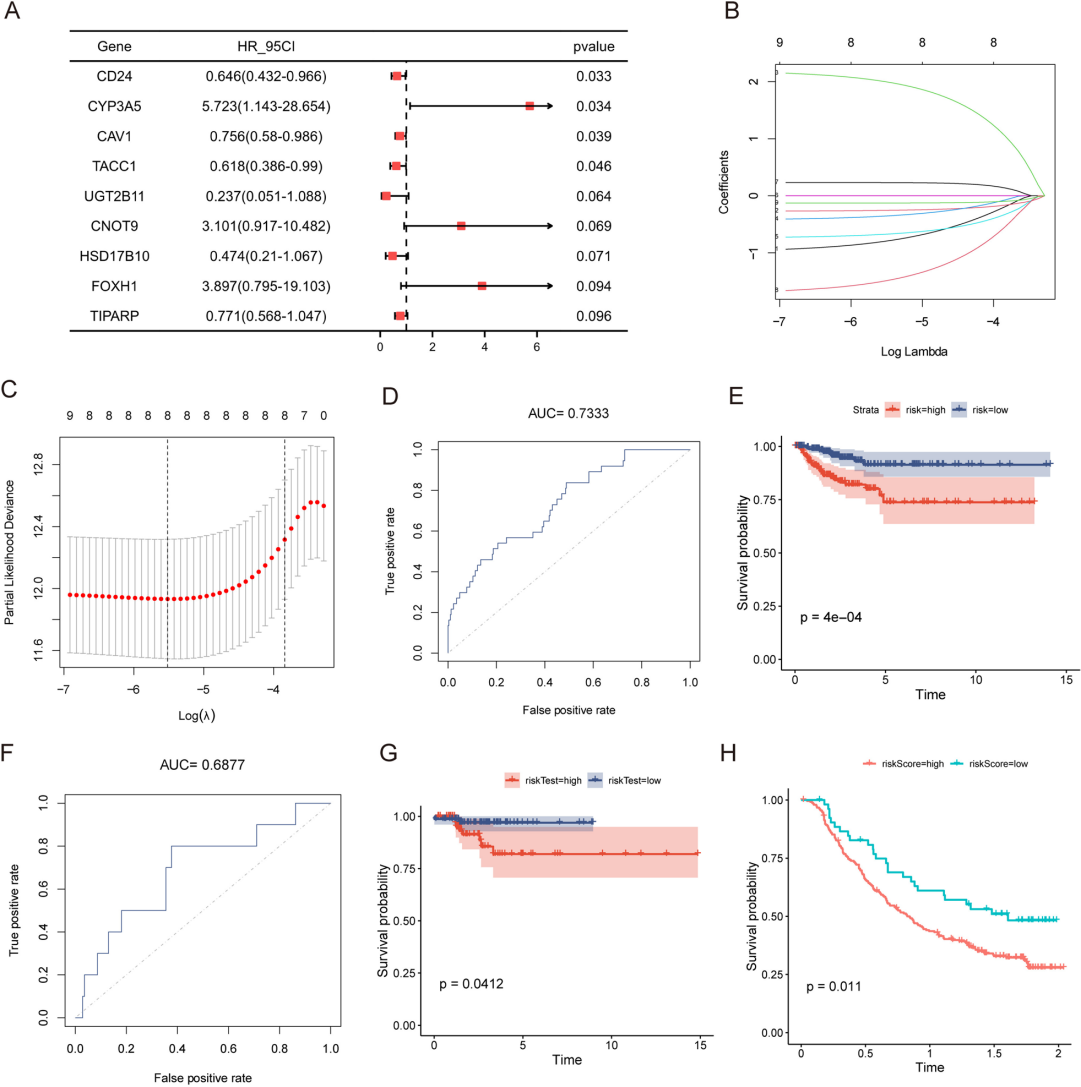
LASSO regression model. **A** Univariate Cox regression analysis. **B** Coefficient distribution plot of eight key genes related to PFI, where each line represents a gene and the ends of these genes point to a vertical coordinate, which is the coefficient. LASSO calculates a coefficient for each gene. **C** Feature selection analysis plot of the key genes. The parameter corresponding to the dashed line on the left is lambda.min (the number above is “8” indicates that the coefficients of eight genes can be retained). **D** ROC curve of training set based on risk score versus survival state. **E** Survival curve for high and low risk groups in the training set. **F** ROC curve of the validation set based on risk scores versus survival status. **G** Survival curve of high and low risk groups in the validation set. **H** Survival curves for high and low risk groups in the immunotherapy dataset. LASSO: Least absolute shrinkage and selection operator; ROC: Receiver operating characteristic; ERDEGs: estrogen-related differentially expressed genes

We divided the 496 patients in the TCGA-THCA dataset into training and validation sets by 7:3 (training sets: 348 cases; verification sets: 148 cases), using univariate Cox regression analysis based on the training sets, analyzing the relationship between variables and PFI. We obtained a total of nine variables (*P <* 0.1), drew forest charts for demonstration (Fig. 3A), preformed LASSO regression (Fig. 3B and C), and filtered eight key genes (UGT2B11, CD24, CYP3A5, TIPARP, TACC1, CNOT9, HSD17B10, and CAV1) associated with PFI. We then established the LASSO regression model based on these eight genes in the training set.

$$\mathrm{Riskscore }=\mathrm{ UGT}2\mathrm{B}11* -0.79669+\mathrm{ CD}24* -0.25591+\mathrm{ CYP}3\mathrm{A}5* 2.00033+\mathrm{ TIPARP}* -0.35507+\mathrm{TACC}1* -0.67401+\mathrm{ CNOT}9* 0.23137+\mathrm{ HSD}17\mathrm{B}10* -1.49204+\mathrm{ CAV}1* -0.12741$$

A validation set was used for authentication. In the training dataset, we plotted the ROC curve for risk scores and their association with PFI status. The area under the curve (AUC) was 0.7333 (Fig. 3D), and we also generated a survival curve for the high- and low-risk groups, yielding a *p*-value of 4e-04 (Fig. 3E). These results demonstrate that the LASSO regression prediction model, established using the eight key genes, can effectively assess the PFI of patients. In the validation dataset, we similarly plotted the ROC curve for risk scores and their correlation with PFI status. The AUC was 0.6877 (Fig. 3F), and the survival curve for the high- and low-risk groups showed a *p*-value of 0.0412 (Fig. 3G). These findings reaffirm the model's ability to accurately evaluate PFI, validating its predictive capability. Therefore, the LASSO regression model established by the eight key genes can well assess patient PFI. We also evaluated the total survival time of patients in the high-risk group using immunotherapy data, which was significantly shorter than that of patients in the low-risk group (Fig. 3H).

### Functional enrichment analysis

**Fig. 4:**
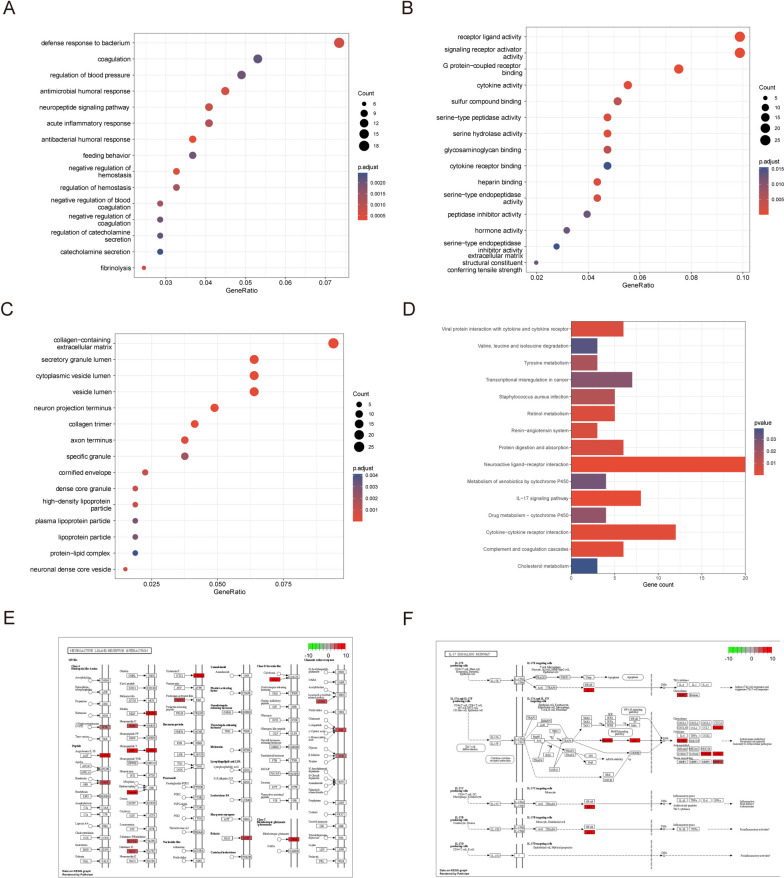
GO and KEGG enrichment analysis. **A** GO enrichment analysis bubble plot for biological processes (BP) showing number of genes vs. GO term; node size indicates number of genes enriched, node color indicates padj. **B** GO enrichment analysis bubble plot for molecular function (MF) showing number of genes vs. GO term; node size indicates number of genes enriched, node color indicates padj. **C** GO enrichment analysis bubble plot for cell components (CC) showing number of genes vs. GO term; node size indicates number of genes enriched, node color indicates padj. **D** KEGG enrichment analysis bar graph showing the number of genes for each KEGG term; color indicates the *P*-value. **E**, **F** Neuroactive ligand–receptor interaction and pathway of the IL-17 signaling pathway [15–17]. GO: Gene Ontology; BP: Biological Processes; MF: Molecular Function; CC: Cell Components; KEGG: Kyoto Encyclopedia of Genes and Genomes

Risk scores were calculated based on coefficients from the LASSO–Cox regression, and all patients with THCA were divided into high- and low-risk groups according to their median. We examined the BP, MF, CC, and pathways (P) involved in the ERDEGs in the high- and low-risk groups. These key genes mostly affect BPs such as antibacterial humoral, coagulation, fibrinolysis, and acute inflammatory responses (Fig. 4A; Table S1). They also affect MF-like receptor–ligand activity, signaling receptor activator activity, G protein-coupled receptor binding, and cytokine activity (Fig. 4B; Table S2). Moreover, they affect the CC-like collagen-containing extracellular matrix, neuron projection term matrix, neuron projection terminus, secretory granule lumen, and cytoplasmic vesicle lumen (Fig. 4C; Table S3), as well as signaling pathways such as the neuroactive ligand–receptor interaction, IL-17 signaling pathway, cytokine–cytokine receptor interaction, complement, and coagulation cascades (Fig. 4D; Table S4). We also identified significantly enriched pathways of neuroactive ligand–receptor interaction and the IL-17 signaling pathway [15–17] (Fig. 4E and F).

### GSEA

**Fig. 5:**
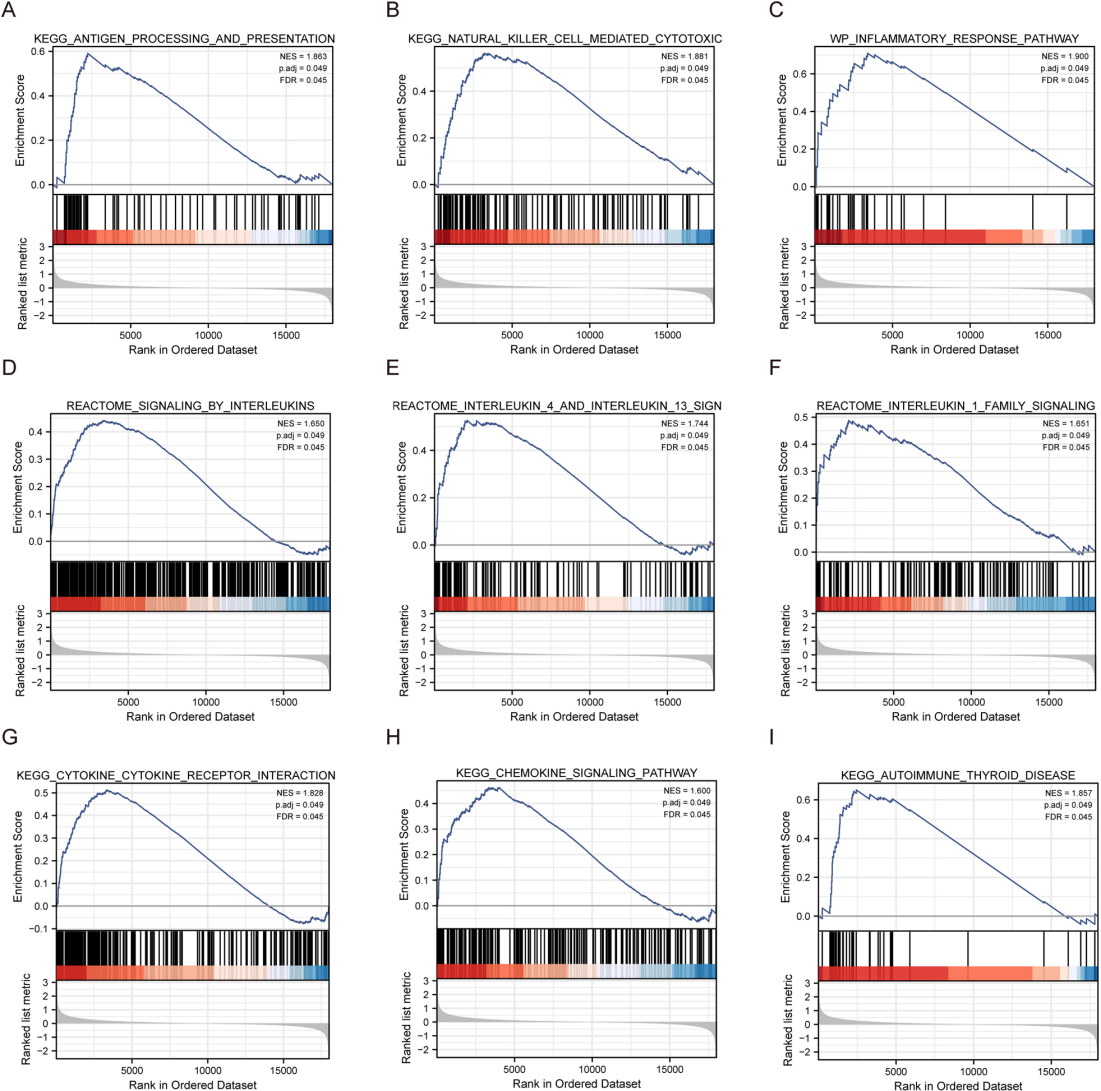
GSEA enrichment analysis. **A**–**I** KEGG ANTIGEN PROCESSING AND PRESENTATION, KEGG NATURAL KILLER CELL MEDIATED CYTOTOXICITY, WP INFLAMMATORY RESPONSE PATHWAY, RE ACTOME SIGNALING BY INTERLEUKINS, REACTOME INTERLEUKIN 4 AND INTE RLEUKIN 13 SIGNALING, REACTOME INTERLEUKIN 1 FAMILY SIGNALING, KEGG CYTOKINE RECEPTOR INTERACTION, KEGG CHEMOKINE SIGNALING PATHWAY, and KEGG AUTOIMMUNE THYROID DISEASE were significantly enriched in the high-risk group. KEGG: Kyoto Encyclopedia of Genes and Genomes

By comparing the high- and low-risk groups, GSEA was used to identify the molecular pathways of ERDEGs that affect prognosis (Table 2). Antigen processing and presentation (Fig. 5A), natural killer cell-mediated cytotoxicity (Fig. 5B), inflammatory response pathway (Fig. 5C), reactome signaling by interleukins (Fig. 5D), reactome interleukin 4 and interleukin 13 signaling (Fig. 5E), reactome interleukin 1 family signaling (Fig. 5F), cytokine receptor interaction (Fig. 5G), chemokine signaling pathway (Fig. 5H), and autoimmune thyroid disease (Fig. 5I) biological functions were significantly enriched in the high-risk group.

**Table 2:** Gene set enrichment analysis

ID	NES	p.adjust
REACTOME_NEUTROPHIL_DEGRANULATION	1.98801794	0.04943101
REACTOME_SIGNALING_BY_INTERLEUKINS	1.65023093	0.04943101
NABA_SECRETED_FACTORS	1.65132116	0.04943101
REACTOME_EXTRACELLULAR_MATRIX_ORGANIZATION	1.74875177	0.04943101
NABA_ECM_REGULATORS	1.95330908	0.04943101
KEGG_CYTOKINE_CYTOKINE_RECEPTOR_INTERACTION	1.82767481	0.04943101
REACTOME_KERATINIZATION	1.93027521	0.04943101
REACTOME_INTERFERON_SIGNALING	1.70414715	0.04943101
NABA_ECM_AFFILIATED	1.70801112	0.04943101
WP_CHEMOKINE_SIGNALING_PATHWAY	1.57964021	0.04943101
KEGG_CHEMOKINE_SIGNALING_PATHWAY	1.59981557	0.04943101
REACTOME_TOLL_LIKE_RECEPTOR_CASCADES	1.6401511	0.04943101
REACTOME_CELL_SURFACE_INTERACTIONS_AT_THE_VASCULAR_WALL	1.96439752	0.04943101
REACTOME_INTERLEUKIN_1_FAMILY_SIGNALING	1.65087184	0.04943101
KEGG_CELL_ADHESION_MOLECULES_CAMS	1.65415851	0.04943101
REACTOME_IMMUNOREGULATORY_INTERACTIONS_BETWEEN_A_LYMPHOID_AND_A_NON_LYMPHOID_CELL	2.23279633	0.04943101
REACTOME_FORMATION_OF_THE_CORNIFIED_ENVELOPE	1.92979623	0.04943101
KEGG_NATURAL_KILLER_CELL_MEDIATED_CYTOTOXICITY	1.88112564	0.04943101
REACTOME_ANTIGEN_PROCESSING_CROSS_PRESENTATION	1.62170743	0.04943101
REACTOME_INTERLEUKIN_4_AND_INTERLEUKIN_13_SIGNALING	1.74429783	0.04943101

### Immuno-infiltration analysis

**Fig. 6:**
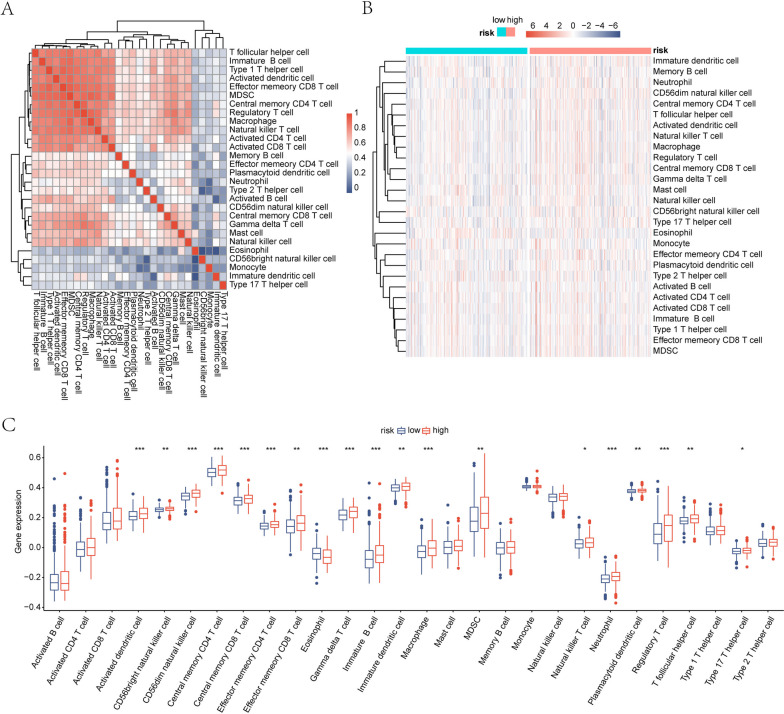
ssGSEA immuno-infiltration analysis. **A** Heat map of immune cell correlations; red represents positive correlations and blue represents negative correlations. **B** Heat map of differences in immune infiltration abundance between high- and low-risk groups; red represents high expression, blue represents low expression, blue annotated bars indicate low risk groups, and pink annotated bars indicate high risk groups. **C** Box plot of differences in immune infiltration abundance between high and low risk groups; the horizontal axis indicates immune cells and the vertical axis indicates immune cell infiltration abundance

Furthermore, to assess the differences in immune cell infiltration in the high- and low-risk groups, we calculated the immune cell infiltration scores for each patient with THCA using the ssGSEA algorithm, demonstrating the distribution of immune cell infiltration in each sample (Fig. 6A) and showing the differences in immune cell infiltration in the high- and low-risk groups using heat maps and box plots, respectively (Fig. 6B and C). The results showed that 19 of the 28 immune cells were significantly associated, suggesting that the key ERDEGs may be significantly associated with these immune cells and that macrophages are significantly upregulated in the high-risk group.

### Construction of a prognostic model of ERDEGs

**Fig. 7:**
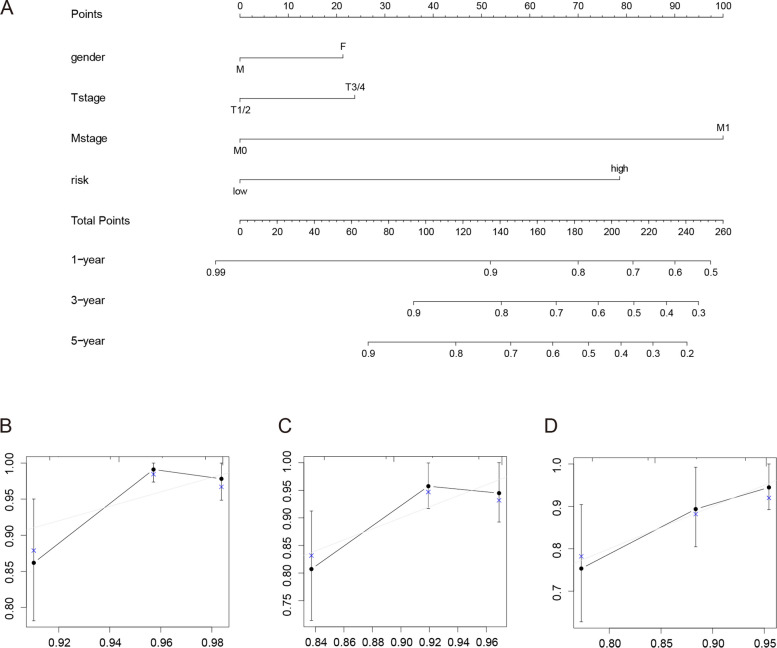
Predictive model. **A** Nomogram plot of thyroid cancer patients based on risk score, T-stage, M-stage, and sex. **B** 1-year calibration plot. **C** 3-year calibration plot. **D** 5-year calibration plot

We created a model based on risk score, T-stage, M-stage, and sex to predict PFI in patients with THCA, plotted a nomogram (Fig. 7A), and tested the model. Figure 7B–D show the calibration plots for patients with THCA at 1-, 3-, and 5-year intervals. The prediction model had some predictive power for the PFI in patients with THCA. For example, a female THCA patient (20.5) with a pathological stage of T3 (21.5), M1 (100), and a higher risk score (79.5) would obtain 221.5 points. Her 1-, 3-, and 5-year PFIs were 66%, 45%, and 31%, respectively.

### Validation of prognostic genes

**Fig. 8:**
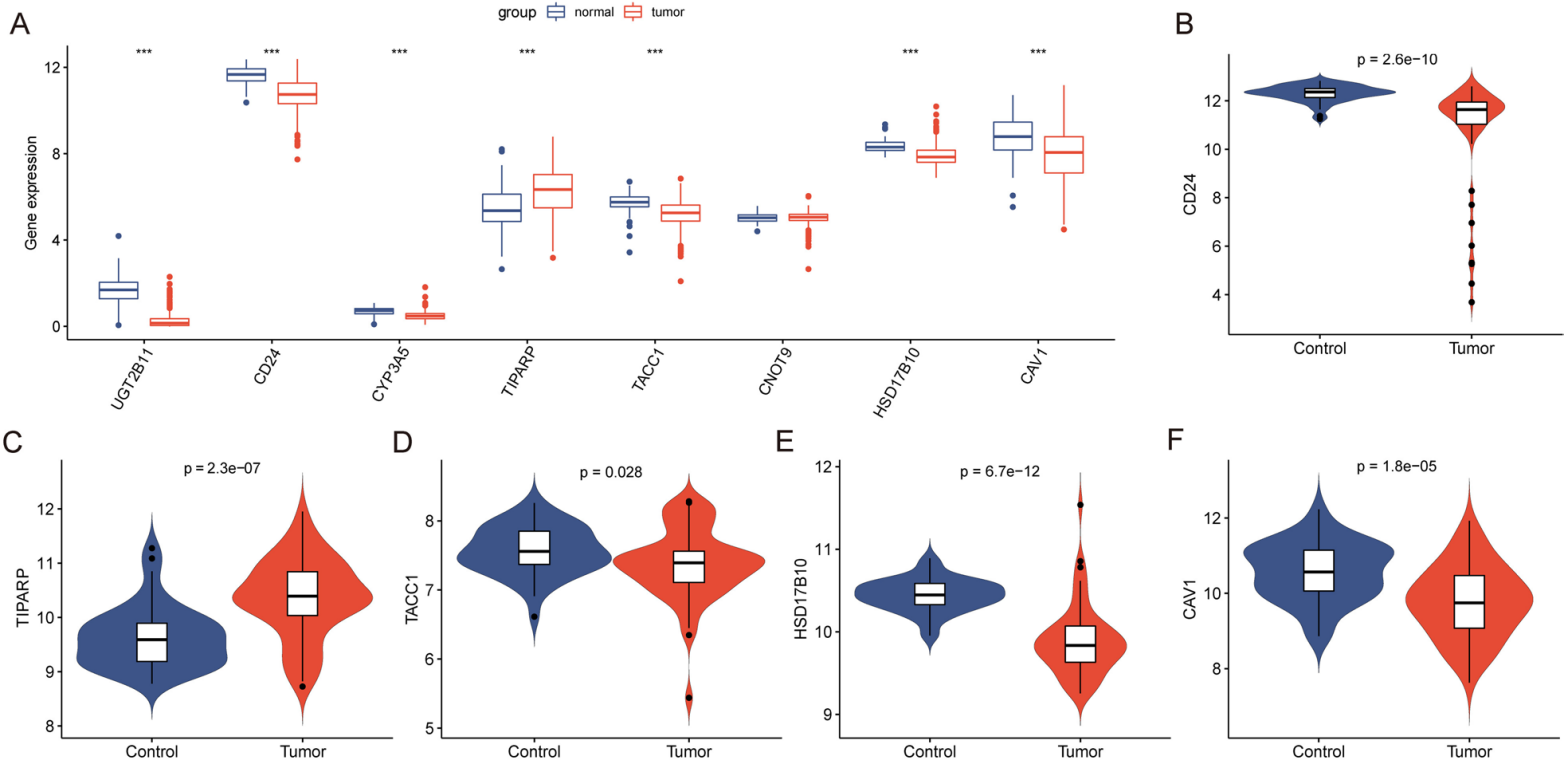
GEO database validation. **A** Differences in expression of key genes between normal and thyroid cancer patients in TCGA; blue represents the normal group and red represents the tumor group. **B**–**F** Differences in expression of key genes between normal and thyroid cancer patients in the GSE33630 dataset; blue represents the normal group and red represents the tumor group. GEO: Gene Expression Omnibus

The key ERDEGs were significantly differentially expressed at the mRNA level between normal individuals and patients with THCA in TCGA (Fig. 8A), and the GSE33630 dataset showed significant differences in CD24, CAV1, TACC1, TIPARP, and HSD17B10 between the normal and patients with THCA groups (Fig. 8B–F). Notably, TIPARP was highly expressed in the tumor relative to the normal group expression, and CD24, TACC1, HSD17B10, and CAV1 were less expressed in the tumor relative to the normal group. We selected TCGA and GEO datasets in which CD24, CAV1, TACC1, TIPARP, and HSD17B10 were differentially expressed in both the normal and THCA groups for subsequent analysis.

### The Correlation among ERDEGs, risk score and THCA stages

**Fig. 9:**
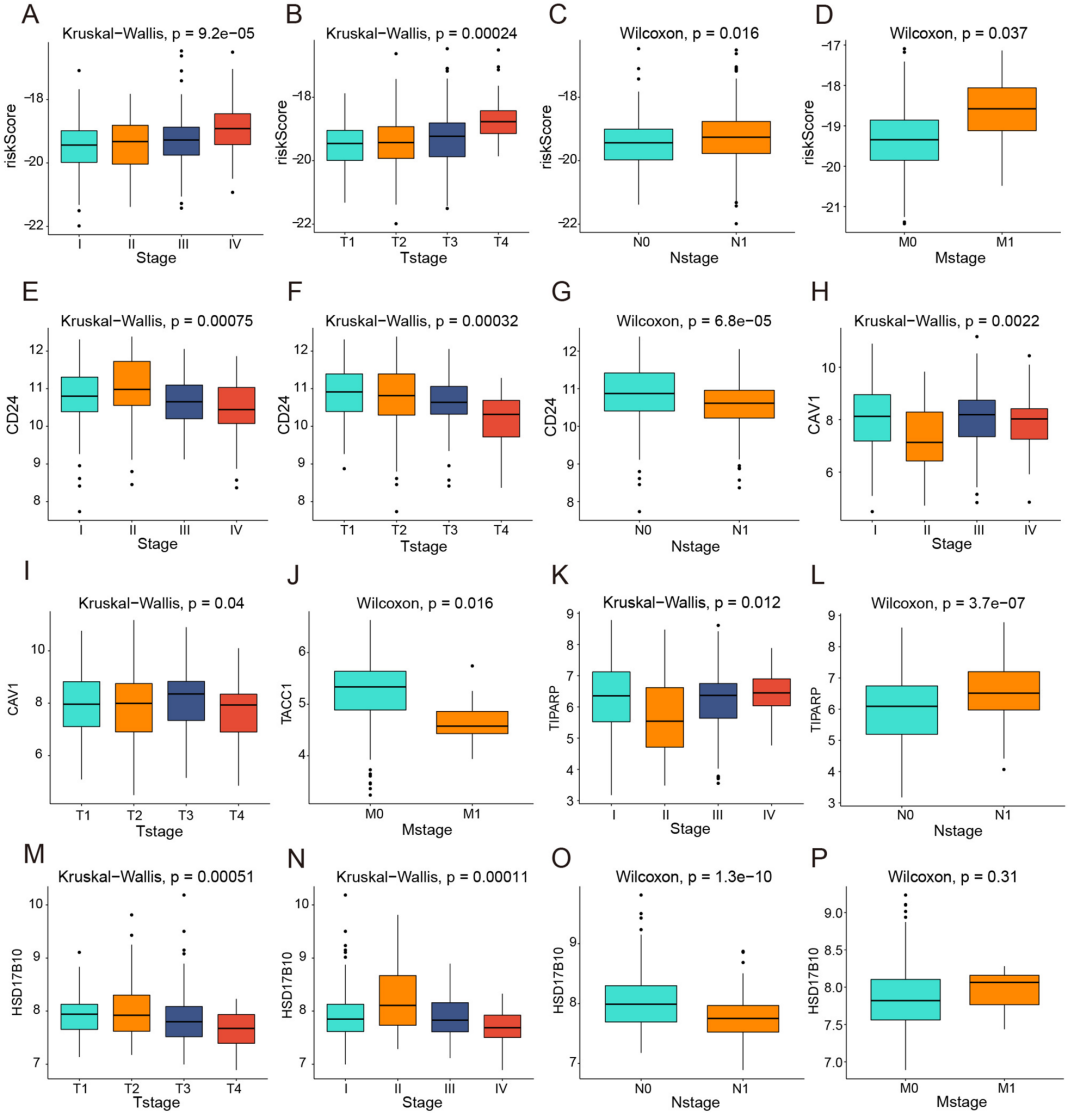
Clinical correlation analysis. **A**–**D** Risk scores were significantly differentially expressed at different pathological stages. **E**–**G** The CD24 gene was significantly associated with the pathological stage, T-stage, and N-stage of patients. **H**, **I** CAV1 expression was significantly associated with the pathological stage and T-stage of thyroid cancer patients. **J** TACC1 expression was significantly associated with the M-stage of thyroid cancer patients. **K**, **L** TIPARP expression was significantly associated with the pathological stage and N-stage of thyroid cancer patients. **M**–**O** HSD17B10 expression was significantly associated with the M-stage of thyroid cancer patients. Significant differences were found between the different pathological stages, T-stage, and N-stage of thyroid cancer patients; no significant differences in the M-stage were observed

Using TCGA (Fig. 9A–P), we examined the relationships among CD24, CAV1, TACC1, TIPARP, and HSD17B10, along with risk score and pathological stages (T, N, and M) in patients with THCA. The risk score was strongly linked to patient pathological (Fig. 9A), T (Fig. 9B), N (Fig. 9C), and M stages (Fig. 9D), with a high-risk score accompanied by a worsening clinical stage. The pathological (Fig. 9E), T (Fig. 9F), and N stages (Fig. 9G) were all significantly linked to CD24 expression, and overall, high CD24 expression was linked to an earlier clinical stage. There was a strong link between the expression of CAV1, patient pathology, and T-stage (Fig. 9H and I). In patients with THCA, TACC1 expression was linked to M-stage, and patients with high TACC1 expression had less distant metastasis (Fig. 9J). In patients with THCA, the expression was linked to both pathological and N stages (Fig. 9K and L). Figure 9M and O shows that the pathological, T, and N stages of thyroid cancer patients were linked to HSD17B10 expression.

### ERDEGs and drug sensitivity analysis 

**Fig. 10:**
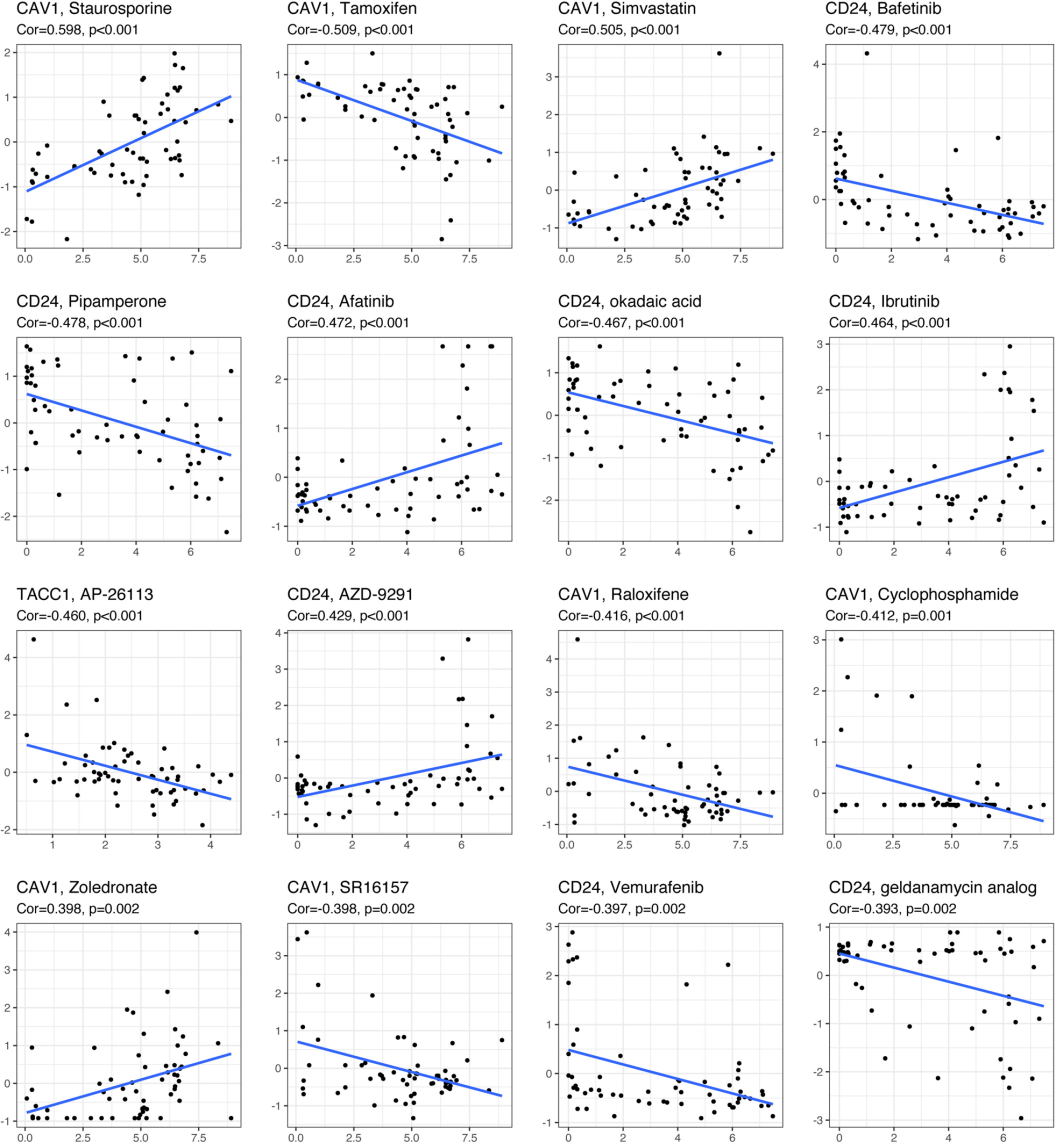
Estrogen-related genes and drug sensitivity analysis. Drug sensitivity analysis of CD24, CAV1, TACC1, TIPARP, and HSD17B10 (CellMiner database)

We analyzed the relationship between the CD24, CAV1, TACC1, TIPARP, and HSD17B10 genes and drug sensitivity using the CellMiner database and identified 16 drugs with the lowest *p*-values for correlation analysis (Fig. 10). Afatinib, AZD-9291, and ibrutinib were more sensitive when CD24 was present, whereas afatinib, ipamperone, okadaic acid, vemurafenib, and the geldanamycin analog were less sensitive when CD24 was present. CD24 negatively correlated with sensitivity to bafetinib, pipamperone, okadaic acid, vemurafenib, and geldanamycin analogs. CAV1 positively correlated with sensitivity to staurosporine, simvastatin, and zoledronate, and CAV1 was negatively correlated with sensitivity to tamoxifen, raloxifene, cyclophosphamide, and SR16157.

To explore the drug sensitivity of patients under different clinical variables, we used drug sensitivity data from the GDSC database to predict the sensitivity of samples with different clinical variables to common anti-cancer drugs. Then we used the Mann-Whitney U test (Wilcoxon rank sum test) to evaluate the difference in sensitivity to different anti-cancer drugs between different groups of patients. Subsequently, we retained the top 20 drugs with relatively large differences in different groups and presented the results. Based on the above results, we found that among the 20 drugs with significant differences, four different clinical variables M stage, N stage, Stage stage and M stage had different sensitivity expressions (Figure S1-S4), which also further emphasized the importance of individualized treatment for tumor patients.

### Validation of key genes by RT–qPCR and IHC

**Fig.11:**
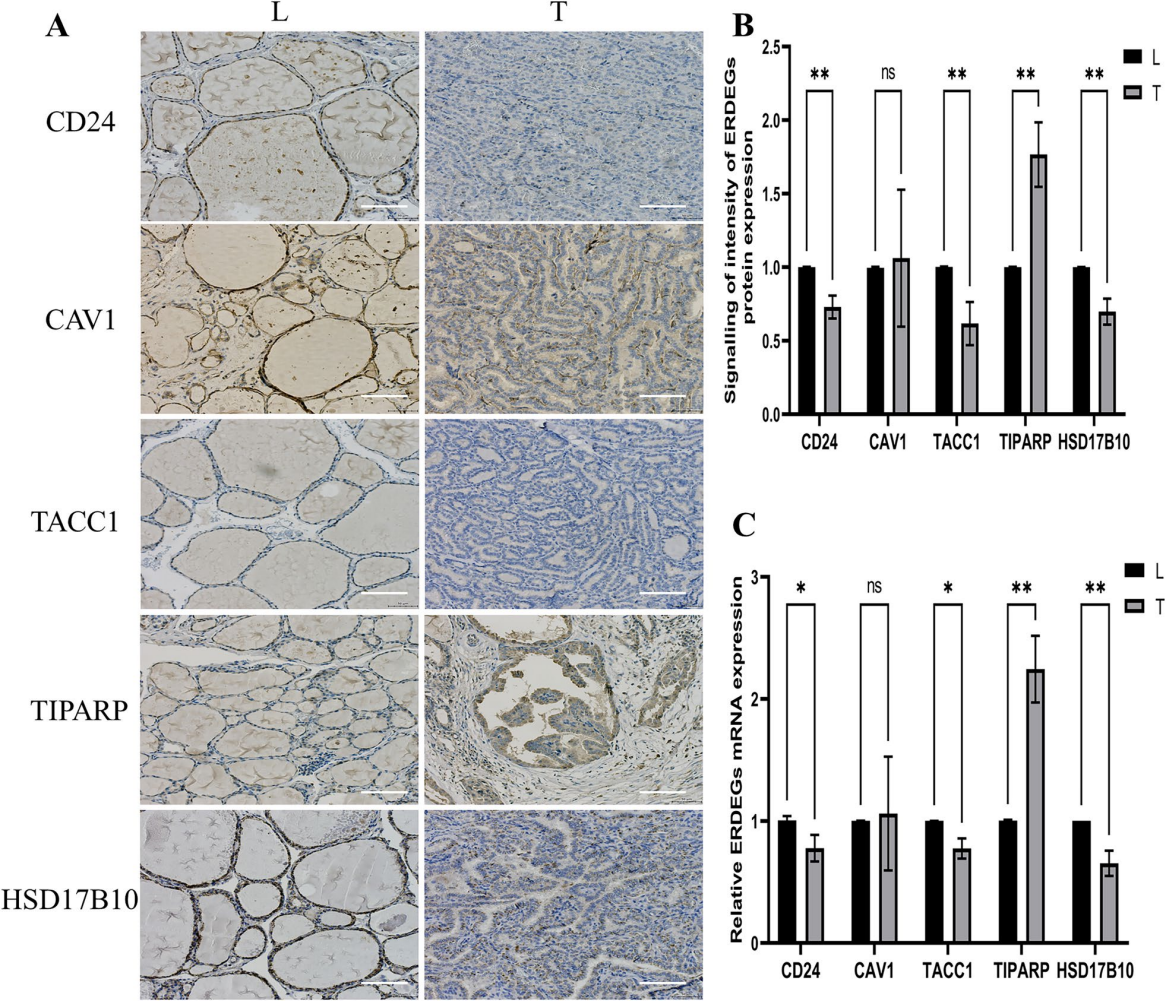
IHC and RT-qPCR in thyroid tissues. **A**, **B** Representative immunohistochemical staining (Bar=100um). **C** RT-qPCR of CD24, CAV1, TACC1, TIPARP, and HSD17B10 in thyroid carcinoma and adjacent tissues. IHC: Immunohistochemistry

RT–qPCR and IHC staining showed that THCA samples from our clinical center expressed CD24, CAV1, TACC1, TIPARP, and HSD17B10. Compared to CD24, TACC1, and HSD17B10, TIPARP was more common in tumors than in normal tissues, but CAV1 was not (Fig. 11).

## Discussion

THCA accounts for 3% of all cancers worldwide, and the number of cases has increased dramatically over the past 30 years. Notably, women experience three to four times as many cases as men; however, the underlying reason remains unclear [18, 19]. The sex-based difference in THCA incidence rate among people aged 0–19 years grows with age and it becomes evident in adolescence. In addition, studies on women with a high rate of THCA have found that the number of pregnancies, age at menarche, natural or artificial menopause, and hormonal contraception are all closely related to the rate of THCA. This suggests that estrogen and its related genes and/or receptors play major roles in the development of THCA [20, 21]. Although estrogen generally exerts its biological effects by binding to estrogen receptors, estrogen receptor mRNA expression in papillary thyroid cancer is lower than that in normal thyroid tissues. Estrogen receptor mRNA expression is not related to sex, age, stage, or lymph node metastasis [22]. In addition, the expression of estrogen and progesterone receptors in pediatric thyroid cancer is not related to sex, American Thyroid Association risk score, persistent structural diseases, or pubertal status. Therefore, studying tumor-related genes that regulate estrogen and progesterone is necessary to explain the higher incidence of female cases in the post-pubertal period [23]. Recent studies have shown that the enrichment analyses of DEGs in papillary thyroid cancer and normal thyroid tissue are mainly enriched in the estrogen response pathway. As the expression of ERGs increases, the likelihood of PTC progression to advanced tumors also increases, and the overall prognosis of patients becomes poor [24]. Therefore, it is important to study how ERGs affect the clinical prognosis, immune invasion, and drug sensitivity of THCA, but not only PTC.

In this study, we performed differential expression analysis to identify 120 ERDEGs, and built a risk model based on 8 of them and validated their reliability. Based on the risk model, differences in enrichment pathways, immune infiltration, and immunotherapy between the high- and low-risk groups were compared. We confirmed that the model was a good way to measure PFI in patients with TCGA-THCA, and examined the overall survival time of patients in the high- and low-risk groups in the immunotherapy dataset. The patients in the high-risk group had significantly shorter survival times than those in the low-risk group. This further confirmed the role of ERDEGs in development and prognosis. We validated the expression of prognosis-related genes in the GEO dataset, and key genes with significant differences in expression and consistent trends (CD24, CAV1, TACC1, TIPARP, and HSD17B10) were subjected to follow-up analysis. We further verified the expression of the five ERDEGs using RT-qPCR and IHC in surgical specimens from patients with THCA.

In particular, CD24 is less common in tumor tissues than in normal tissues. In cases in which CD24 is more common, the clinical stage occurs earlier. This is consistent with previous research on databases. An earlier study [25] showed that THCA cells with low CD24 expression expressed higher levels of stem cell markers and lower levels of thyroid differentiation markers, suggesting that THCA cells with low CD24 expression have higher tumor stemness and may be responsible for tumor recurrence, metastasis, and drug resistance. One study found that THCA cases in TCGA were grouped according to RNA expression of tumor stem cell markers and analyzed for recurrence-free survival between groups, showing that the low CD24 expression group had a significantly worse 12-year recurrence-free survival rate, CD24 participates in the molecular subtype identification of PTC, and high PROM 1, high ALDH 1A 3, and low CD24 in the tall cell variant of papillary thyroid carcinoma have significantly poorer recurrence-free survival rates [26]. In another study, CD24 was low in papillary thyroid cancer, and its expression was negatively correlated with multifocality [27]. In a multicenter study on THCA, CD24 expression was 11 times higher in people under the age of 18 years than in adults [28]. CD24 interacts with the inhibitory receptor Siglec-10 on innate immune cells to achieve immune escape [29], which may be why THCA is more likely to spread in children. Moreover, it is possible that estrogen-mediated downregulation of CD24 expression is responsible for the high failure rate of selective estrogen receptor modulators in breast cancer treatment [30]. Whether there is a relationship between CD24 and the development, recurrence, and non-uptake of iodine in thyroid cancer needs to be explored in further experiments. The drug sensitivity analysis of key genes showed that CD24 was positively correlated with the sensitivity of targeted drugs afatinib, AZD-9291, and ibrutinib, whereas CD24 was negatively correlated with the sensitivity of bafatinib, pipamperone, okadaic acid, vemurafenib, and geldanamycin analog drugs. For late-stage, recurrent, and refractory patients with THCA in clinical practice, we can select sensitive drugs by detecting the expression of CD24 and avoid insensitive drugs.

The expression levels of CAV1 vary among different tumors. Single-cell RNA analysis showed that CAV1 affects the glycolytic activity in pancreatic cancer fibroblasts and is related to the expression of glycolytic enzymes. An increase in glycolysis in cancer-associated fibroblasts and a decrease in CAV1 expression can promote tumor progression [31]. In cervical cancer, expression of CAV1 increases, and the long-term survival rate of patients is reduced [32]. CAV1 is not expressed in normal thyroid tissue but is highly expressed in thyroid cancer, especially in microcarcinoma [33]. In our study, 51.6% of THCA tissues highly expressed CAV1, and its high expression was accompanied by early pathological- and T-stage. This phenomenon may be due to the difference in the stage distribution of cases between TCGA and our clinical study. TCGA has more intermediate and advanced thyroid cancer cases, whereas our study had more early-stage cases. CAV1 promotes tumor angiogenesis and mediates the unlimited proliferation and self-renewal of tumor stem cells at the initiation stage of malignancy [34, 35]. Bioinformatics studies have shown that CAV1 is a common hub gene that plays an important role in the occurrence and development of parathyroid and thyroid follicular adenomas. Enrichment analysis has shown that it is closely related to cell proliferation [36]. Further research is required to explore whether and how CAV1 participates in the early development of THCA. During the metastatic stage of malignant tumors, CAV1 is involved in adhesion movement, loss of nest apoptosis, and autophagy in tumor cells [37, 38]. In addition, CAV1 expression can directly or indirectly promote tumor multidrug resistance [39, 40] and affect the interaction of tumor cells with the mesenchymal microenvironment [41]. However, tumor heterogeneity leads to two-sided CAV1 expression; thus, the role of CAV1 as an oncogene or oncogenic factor is controversial. In-depth studies on the CAV1 signaling pathway and its relationship with tumors are expected to provide new avenues for the diagnosis, treatment, and prevention of related tumors. In our drug sensitivity analysis, we found a negative correlation between CAV1 expression and sensitivity to several estrogen receptor modulators. Whether estrogen receptor modulators can achieve the expected efficacy in clinically low-CAV1-expressing THCA cases with advanced stages and high malignancy requires further investigation. In THCA with high CAV1 expression, staurosporine, simvastatin, and zoledronate can be selected.

In THCA, the higher the TACC1 expression, the lower the occurrence of distant metastasis. TACC1 plays a role in tumor growth by binding to many different complexes, and the downregulation of human TACC1 may alter how polarized cells control mRNA homeostasis and contribute to the development of cancer [42]. Researchers have examined how TACC1 functions as a regulator in breast and ovarian cancers; however, there has not been a separate report on THCA. Our bioinformatics analysis showed that TACC1 expression in THCA was lower than that in paracrine tissue. In 2021, a case report showed that patients with low-grade primary spinal gliomas developed rapid and malignant transformations after pregnancy and delivery. FGFR1 and TACC1 genes were fused with FGFR1 activation, and the function of the tumor suppressor gene STED2 was lost in high-throughput sequencing. We speculated that disease progression and malignant transformation are related to the alteration of TACC1 function by high estrogen levels during pregnancy [43]. In addition, drug sensitivity analysis showed that the expression of TACC1 negatively correlated with the sensitivity to AP-26113, which is expected to be highly effective in cases of THCA with low TACC1 expression. The exact mechanism and whether it has any effect need to be further explored, and the best treatment for patients with ATC is multikinase inhibitors (MKIs). However, the efficacy and toxicity of MKIs are heterogeneous and difficult to predict before treatment initiation. Even after the occurrence of serious adverse events, treatment needs to be interrupted [44]. For this group of patients, we can consider testing the expression of CD24, CAV1, and TACC1, and select the corresponding sensitive drugs based on the test results.

TIPARP is a member of the polyadenosine diphosphate ribose polymerase family and is involved in the cellular stress response and DNA damage repair [45]. The less active TIPARP in breast cancer, the less likely patient survival [46]. TIPARP directly targets HIF-1α and recruits E3 ubiquitin-protein ligase to promote the degradation and inactivation of HIF-1α to inhibit tumor growth [47]. Estradiol induces the expression of TIPARP in breast cancer cells via ER-α, TIPARP is a negative feedback regulator of ER-α that inhibits the transcriptional activity and expression of ER-α in breast cancer cells and reduces its recruitment to target genes, and TIPARP knockdown increases the transcriptional activity and expression of ER-α to promote estradiol-induced cell proliferation [48]. However, the TIPARP inhibitor RBN-2397 has been shown to induce STAT1 phosphorylation and upregulates type I IFN signaling, leading to a durable and complete regression of NCI-H1373 cell xenograft tumors. Although TIPARP exhibits opposing effects in different tumor cells, in general, TIPARP is involved in the regulation of tumor cells by estrogen and is a potential target for the study of anti-tumor drugs. The role and mechanism of TIPARP in tumors remain unclear, especially in THCA, where the mechanism of action of TIPARP has not been reported. Our study showed that the expression of TIPARP is significantly higher in tumors than in paraneoplastic tissues and that the expression of TIPARP correlates significantly with the pathological and N stages of patients with THCA.

HSD17B10 is an enzyme found in mitochondria that converts 17-estradiol into estrone, a weaker chemical [49]. Estradiol is thought to play an important role in normal aging [50, 51], where it regulates mitochondrial homeostasis by reducing oxidative stress and preventing cytochrome release and apoptosis to maintain the mitochondrial structure. When estradiol is oxidized to the weaker estrone, this estrogen is controlled using proteomic data and bioinformatics on thyroid tissue, and HSD17B10 was used to identify possible markers for thyroid follicular tumors and carcinomas [52]. Overexpression of HSD17B10 in pheochromocytoma cells leads to abnormal cell growth in the laboratory and in the body, and high levels of HSD17B10 are linked to poor patient prognosis. In prostate cancer, HSD17B10 is administered in combination with steroids and produces dihydrotestosterone in the absence of testosterone [53], which is a different way of producing androgens. In colorectal cancer, high HSD17B10 expression is associated with improved overall survival [54]. This study showed that the expression of HSD17B10 is significantly lower in paracancerous tissue than in tumor tissue and that the expression of HSD17B10 correlates with the pathological, T, and N stages of patients with THCA. HSD17B10 is closely related to the conversion of estradiol, and the relationship between the two and the development of THCA requires further investigation.

In this study, we examined all the ERGs linked to the development of THCA. We created and tested a risk score model for the TCGA-THCA cohort based on the risk score, T-stage, M-stage, and sex. The results showed that the risk score model accurately predicted PFI at 1, 3, and 5 years for patients with THCA. In addition, GSEA showed that these ERDEGs may be involved in the immune regulation of THCA. Immune infiltration analysis showed that compared with the low-risk group, in the high-risk group, significant differences in 19 types of immune cells were observed, and macrophages were significantly upregulated. The immune system plays a key role in tumor development. Tumor cells are recognized and destroyed through immune mechanisms, whereas tumor immune escape results in the clonal growth of tumor cells and the development of tumors. Immune cells play dual roles in the growth and progression of THCA. Tumor-infiltrating immune cells can perform both anti-tumor and pro-tumor functions in THCA, and a number of soluble factors (cytokines, chemokines, angiogenic factors, and lymphangiogenic factors) released by immune cells mediate the pro-tumor and anti-tumor effects of immune cells in THCA. Tumor-associated macrophages (TAMs) are the most studied immune cells. TAMs are distributed differently in different subtypes of THCA, with low infiltration of TAMs in PTC, a high density of TAMs in THCA, and a poor prognosis [55, 56]. In vitro studies have reported that TAMs promote the invasion of human TC cell lines through CXCL8 and IL-8 secretion [57]. A study analyzing the GSE129562 dataset identified 729 DEGs between T1aN1b or T3N1b and the corresponding normal thyroid tissue. Among these, 138 DEGs were identified as immune-related genes, indirectly suggesting that immunity is involved in the progression of THCA [58]. This indirectly validated part of the study on immune infiltration analysis.

However, the above conclusions were derived from bioinformatics analyses. When we screened key genes, because the number of genes obtained by *P <* 0.05 was too small, we used a threshold of *P <* 0.1 to expand the potential genes. Further, we used LASSO regression when building prognostic models because our study aimed to conduct preliminary exploratory analysis to identify more potential biomarkers and trends, which was supported by the subsequent preliminary model validation. Some published literature used the same P-threshold and modeling analysis methods, but in future analyses, we hope to implement a stricter P-threshold and choose more appropriate modeling analysis methods. We used RT-qPCR and IHC to evaluate the expression of ERDEGs in human THCA and paracancerous tissues from the same patients. Further studies are required to elucidate the cancer phenotypes associated with these key genes. Also, the molecular mechanisms underlying the relationship between estrogen and ERDEGs are poorly understood. The mechanisms by which ERDEGs regulate the proliferation, invasion, metastasis, and iodine uptake of THCA remain poorly defined. In future work, we will study the correlations among estrogen, ERGs, and biological characteristics of THCA, such as tumor microenvironment drug sensitivity, in order to better explain the reason for the high incidence rate of female thyroid cancer and improve the treatment options.

## Conclusion

In conclusion, we developed and tested a risk scoring system for ERGs based on TCGA datasets for THCA patient prognosis assessment and risk stratification, and set up a histone chart model to predict progression-free intervals of 1, 3, and 5 years. We identified eight ERDEGs that may be potential targets for understanding the biological mechanisms of THCA. GSEA and tumor immune invasion analyses also showed that ERGs may be involved in immunomodulatory and autoimmune thyroid diseases. These results provide new insights for THCA research, primarily molecular evidence for the critical role of ERGs in regulating the THCA immune microenvironment, and therapeutic response.

### Supplementary Information

**Additional file 1: Table S1.** GO BP enrichment analysis. Legend:GO BP enrichment analysis.

**Additional file 2: Table S2.** GO MF enrichment analysis. Legend:GO MF enrichment analysis.

**Additional file 3: Table S3.** GO CC enrichment analysis. Legend:GO CC enrichment analysis.

**Additional file 4: Table S4.** KEGG enrichment analysis. Legend:KEGG enrichment analysis.

**Additional file 5: Figure S1.** Drug Sensitivity Analysis of M0 and M1. Group comparison plots of the sensitivity analysis results of drugs Erlotinib (A), BI.D1870 (B), BMS.708163 ©, Lapatinib (D), Rapamycin (E), Mitomycin.C (F), AKT.inhibitor.VIII (G), BX.795 (H), XMD8.85 (I), AZD.0530 (J), Bortezomib (K), DMOG (L), BIRB.0796 (M), Temsirolimus (N), CGP.60474 (O), AP.24534 (P), Etoposide (Q), QS11 ®, LFM.A13 (S) and Cisplatin (T) for M0 and M1 in disease samples from the TCGA-THCA dataset based on the GDSC database. THCA, Thyroid Cancer; TCGA, The Cancer Genome Atlas. ***p value < 0.001, which is highly statistically significant. Yellow represents M0, green represents M1.

**Additional file 6: Figure S2.** Drug Sensitivity Analysis of N0 and N1. Group comparison plots of the sensitivity analysis results of drugs MK.2206 (A), AZD8055 (B), BIBW2992 ©, X17.AAG (D), PLX4720 (E), Vorinostat (F), Sorafenib (G), AZD6244 (H), ABT.888 (I), AG.014699 (J), JNK.Inhibitor.VIII (K), Nutlin.3a (L), GDC0941 (M), Metformin (N), SL.0101.1 (O), Thapsigargin (P), IPA.3 (Q), CHIR.99021 ®, WO2009093972 (S) and KU.55933 (T) for N0 and N1 in disease samples from the TCGA-THCA dataset based on the GDSC database. THCA, Thyroid Cancer; TCGA, The Cancer Genome Atlas. ***p value < 0.001, which is highly statistically significant. Yellow represents N0, green represents N1.

**Additional file 7: Figure S3.** Drug Sensitivity Analysis of Stage I Stage II Stage III and Stage IV. Group comparison plots of the sensitivity analysis results of drugs KU.55933 (A), Etoposide (B), BIBW2992 (C), PF.562271 (D), PD.0332991 (E), Bosutinib (F), Erlotinib (G), Doxorubicin (H), Bleomycin (I), NU.7441 (J), Dasatinib (K), AMG.706 (L), Roscovitine (M), BI.2536 (N), Tipifarnib (O), A.443654 (P), AZD6244 (Q), CI.1040 (R), Gemcitabine (S) and CHIR.99021 (T) for Stage I, Stage II, Stage III and Stage IV in disease samples from the TCGA-THCA dataset based on the GDSC database. THCA, Thyroid Cancer; TCGA, The Cancer Genome Atlas. *** indicates *p* value < 0.001, which is highly statistically significant. Yellow represents Stage I, blue represents Stage II, purple represents Stage III, green represents Stage IV.

**Additional file 8: Figure S4.** Drug Sensitivity Analysis of T1 T2 T3 and T4. Group comparison plots of the sensitivity analysis results of drugs KU.55933 (A), MK.2206 (B), Vorinostat (C), JNK. Inhibitor. VIII (D), Metformin (E), IPA.3 (F), PLX4720 (G), SL.0101.1 (H), SB.216763 (I), NU.7441 (J), Rapamycin (K), Sorafenib (L), WO2009093972 (M), GSK269962A (N), JNJ.26854165 (O), ABT.888 (P), AZD6244 (Q), ATRA (R), PF.02341066 (S) and Elesclomol (T) for T1, T2, T3 and T4 in disease samples from the TCGA-THCA dataset based on the GDSC database. THCA, Thyroid Cancer; TCGA, The Cancer Genome Atlas. *** indicates *p* value < 0.001, which is highly statistically significant. Yellow represents T1I, blue represents T2, purple represents T3, green represents T4.

## Data Availability

The data that support the findings of this study are publicly available from TCGA (https://portal.gdc.cancer.gov/projects/TCGA-THCA) , the GSE33630 dataset (https://www.ncbi.nlm.nih.gov/geo/query/acc.cgi?acc=GSE33630), and the GO database(https://www.informatics.jax.org/vocab/gene_ontology/GO:0019731), Other GO accession numbers can be found in additional files Table S1-3; estrogen-related genes were selected from https://www.gsea-msigdb.org/gsea/index.jsp.

## Abbreviations

THCA: Thyroid cancer
ERGs: Estrogen-related genes
GEO: Gene Expression Omnibus
TCGA: The Cancer Genome Atlas
LASSO: Least absolute shrinkage and selection operator
DEGs: Differentially expressed genes
PFI: Progression-free intervals
IHC: Immunohistochemistry
ERDEGs: Estrogen-related differentially expressed genes
PTC: Papillary thyroid cancer
TPM: Total productive maintenance
GSEA: Gene set enrichment analysis
GO: Gene ontology
BP: Biological processes
MF: Molecular functions
CC: Cellular components
KEGG: Kyoto Encyclopedia of Genes and Genomes
RT: Reverse transcription
ROC: Receiver operating characteristic
AUC: Area under the curve
TKIs: Multikinase inhibitors
TAMs: Tumor-associated macrophages
GDSC: Genomics of Drug Sensitivity in Cancer
