# Supplementary material for: Estrogen-related genes for thyroid cancer prognosis, immune infiltration, staging, and drug sensitivity
Source: BMC Cancer. 2023 Oct 31;23:1048. doi: 10.1186/s12885-023-11556-0 (PMC10619281; doi:10.1186/s12885-023-11556-0)
Supplement: Supplementary file 1 — Additional file 1: Table S1. GO BP enrichment analysis. Legend:GO BP enrichment analysis. [file 12885_2023_11556_MOESM1_ESM.docx]

Additional file1：

Title:Table S1 GO BP enrichment analysis

Legend:GO BP enrichment analysis

| ID | Description | p.adjust |
| --- | --- | --- |
| GO:0019731 | antibacterial humoral response | 0.00029804 |
| GO:1900047 | negative regulation of hemostasis | 0.00047544 |
| GO:0019730 | antimicrobial humoral response | 0.00062993 |
| GO:0042730 | fibrinolysis | 0.00062993 |
| GO:0042742 | defense response to bacterium | 0.00066237 |
| GO:0007218 | neuropeptide signaling pathway | 0.00080376 |
| GO:0002526 | acute inflammatory response | 0.00114298 |
| GO:1900046 | regulation of hemostasis | 0.00133623 |
| GO:0030195 | negative regulation of blood coagulation | 0.00133717 |
| GO:0050819 | negative regulation of coagulation | 0.00199193 |
| GO:0007631 | feeding behavior | 0.00199193 |
| GO:0008217 | regulation of blood pressure | 0.00199193 |
| GO:0050817 | coagulation | 0.00214703 |
| GO:0050433 | regulation of catecholamine secretion | 0.00214703 |
| GO:0050432 | catecholamine secretion | 0.00244965 |
| GO:0030198 | extracellular matrix organization | 0.00244965 |
| GO:0043062 | extracellular structure organization | 0.00244965 |
| GO:0045229 | external encapsulating structure organization | 0.00250181 |
