# Supplementary material for: Estrogen-related genes for thyroid cancer prognosis, immune infiltration, staging, and drug sensitivity
Source: BMC Cancer. 2023 Oct 31;23:1048. doi: 10.1186/s12885-023-11556-0 (PMC10619281; doi:10.1186/s12885-023-11556-0)
Supplement: Supplementary file 2 — Additional file 2: Table S2. GO MF enrichment analysis. Legend:GO MF enrichment analysis. [file 12885_2023_11556_MOESM2_ESM.docx]

Additional file 2:

Title:Table S2 GO MF enrichment analysis

Legend:GO MF enrichment analysis

| ID | Description | p.adjust |
| --- | --- | --- |
| GO:0048018 | receptor ligand activity | 5.48E-06 |
| GO:0030546 | signaling receptor activator activity | 5.48E-06 |
| GO:0001664 | G protein-coupled receptor binding | 5.48E-06 |
| GO:0005125 | cytokine activity | 0.00070786 |
| GO:0008236 | serine-type peptidase activity | 0.00158033 |
| GO:0017171 | serine hydrolase activity | 0.00158033 |
| GO:0008201 | heparin binding | 0.00158033 |
| GO:0004252 | serine-type endopeptidase activity | 0.00214092 |
| GO:1901681 | sulfur compound binding | 0.00489589 |
| GO:0005539 | glycosaminoglycan binding | 0.00489589 |
| GO:0030020 | extracellular matrix structural constituent conferring tensile strength | 0.01221236 |
| GO:0030414 | peptidase inhibitor activity | 0.01254737 |
| GO:0005179 | hormone activity | 0.01254737 |
| GO:0005126 | cytokine receptor binding | 0.01551961 |
| GO:0004867 | serine-type endopeptidase inhibitor activity | 0.01551961 |
| GO:0042379 | chemokine receptor binding | 0.01657948 |
| GO:0048020 | CCR chemokine receptor binding | 0.01669555 |
| GO:0030348 | syntaxin-3 binding | 0.01760248 |
| GO:0005201 | extracellular matrix structural constituent | 0.01880435 |
| GO:0008195 | phosphatidate phosphatase activity | 0.01880435 |
