# Supplementary material for: Estrogen-related genes for thyroid cancer prognosis, immune infiltration, staging, and drug sensitivity
Source: BMC Cancer. 2023 Oct 31;23:1048. doi: 10.1186/s12885-023-11556-0 (PMC10619281; doi:10.1186/s12885-023-11556-0)
Supplement: Supplementary file 3 — Additional file 3: Table S3. GO CC enrichment analysis. Legend:GO CC enrichment analysis. [file 12885_2023_11556_MOESM3_ESM.docx]

Additional file 3:

Title:Table S3 GO CC enrichment analysis

Legend:GO CC enrichment analysis

| ID | Description | p.adjust |
| --- | --- | --- |
| GO:0062023 | collagen-containing extracellular matrix | 3.21E-07 |
| GO:0005581 | collagen trimer | 4.70E-06 |
| GO:0044306 | neuron projection terminus | 7.97E-06 |
| GO:0034774 | secretory granule lumen | 0.00015621 |
| GO:0060205 | cytoplasmic vesicle lumen | 0.00015621 |
| GO:0031983 | vesicle lumen | 0.00015621 |
| GO:0043679 | axon terminus | 0.00033034 |
| GO:0098992 | neuronal dense core vesicle | 0.0006621 |
| GO:0031045 | dense core granule | 0.00098739 |
| GO:0034364 | high-density lipoprotein particle | 0.00098739 |
| GO:0001533 | cornified envelope | 0.00099008 |
| GO:0042581 | specific granule | 0.00202474 |
| GO:0034358 | plasma lipoprotein particle | 0.00298023 |
| GO:1990777 | lipoprotein particle | 0.00298023 |
| GO:0032994 | protein-lipid complex | 0.00410913 |
| GO:0035580 | specific granule lumen | 0.00423555 |
| GO:0071682 | endocytic vesicle lumen | 0.00495363 |
| GO:0060200 | clathrin-sculpted acetylcholine transport vesicle | 0.01004327 |
| GO:0060201 | clathrin-sculpted acetylcholine transport vesicle membrane | 0.01004327 |
| GO:0042583 | chromaffin granule | 0.01067 |
