# Supplementary material for: Estrogen-related genes for thyroid cancer prognosis, immune infiltration, staging, and drug sensitivity
Source: BMC Cancer. 2023 Oct 31;23:1048. doi: 10.1186/s12885-023-11556-0 (PMC10619281; doi:10.1186/s12885-023-11556-0)
Supplement: Supplementary file 4 — Additional file 4: Table S4. KEGG enrichment analysis. Legend:KEGG enrichment analysis. [file 12885_2023_11556_MOESM4_ESM.docx]

Additional file 4:

Title:Table S4 KEGG enrichment analysis

Legend:KEGG enrichment analysis

| ID | Description | p.adjust |
| --- | --- | --- |
| hsa04080 | Neuroactive ligand-receptor interaction | 4.30E-05 |
| hsa04657 | IL-17 signaling pathway | 0.00675844 |
| hsa04060 | Cytokine-cytokine receptor interaction | 0.0730582 |
| hsa04610 | Complement and coagulation cascades | 0.0730582 |
| hsa00830 | Retinol metabolism | 0.10144575 |
| hsa04061 | Viral protein interaction with cytokine and cytokine receptor | 0.10144575 |
| hsa04974 | Protein digestion and absorption | 0.10144575 |
| hsa04614 | Renin-angiotensin system | 0.10144575 |
| hsa05150 | Staphylococcus aureus infection | 0.2791903 |
| hsa00350 | Tyrosine metabolism | 0.28406406 |
| hsa00982 | Drug metabolism - cytochrome P450 | 0.35744343 |
| hsa05202 | Transcriptional misregulation in cancer | 0.36527855 |
| hsa00980 | Metabolism of xenobiotics by cytochrome P450 | 0.39175852 |
| hsa00280 | Valine, leucine and isoleucine degradation | 0.4319042 |
| hsa04979 | Cholesterol metabolism | 0.4471221 |
