# Supplementary material for: Estrogen-related genes for thyroid cancer prognosis, immune infiltration, staging, and drug sensitivity
Source: BMC Cancer. 2023 Oct 31;23:1048. doi: 10.1186/s12885-023-11556-0 (PMC10619281; doi:10.1186/s12885-023-11556-0)
Supplement: Supplementary file 5 — Additional file 5: Figure S1. Drug Sensitivity Analysis of M0 and M1. Group comparison plots of the sensitivity analysis results of drugs Erlotinib (A), BI.D1870 (B), BMS.708163 ©, Lapatinib (D), Rapamycin (E), Mitomycin.C (F), AKT.inhibitor.VIII (G), BX.795 (H), XMD8.85 (I), AZD.0530 (J), Bortezomib (K), DMOG (L), BIRB.0796 (M), Temsirolimus (N), CGP.60474 (O), AP.24534 (P), Etoposide (Q), QS11 ®, LFM.A13 (S) and Cisplatin (T) for M0 and M1 in disease samples from the TCGA-THCA dataset based on the GDSC database. THCA, Thyroid Cancer; TCGA, The Cancer Genome Atlas. ***p value < 0.001, which is highly statistically significant. Yellow represents M0, green represents M1. [file 12885_2023_11556_MOESM5_ESM.docx]

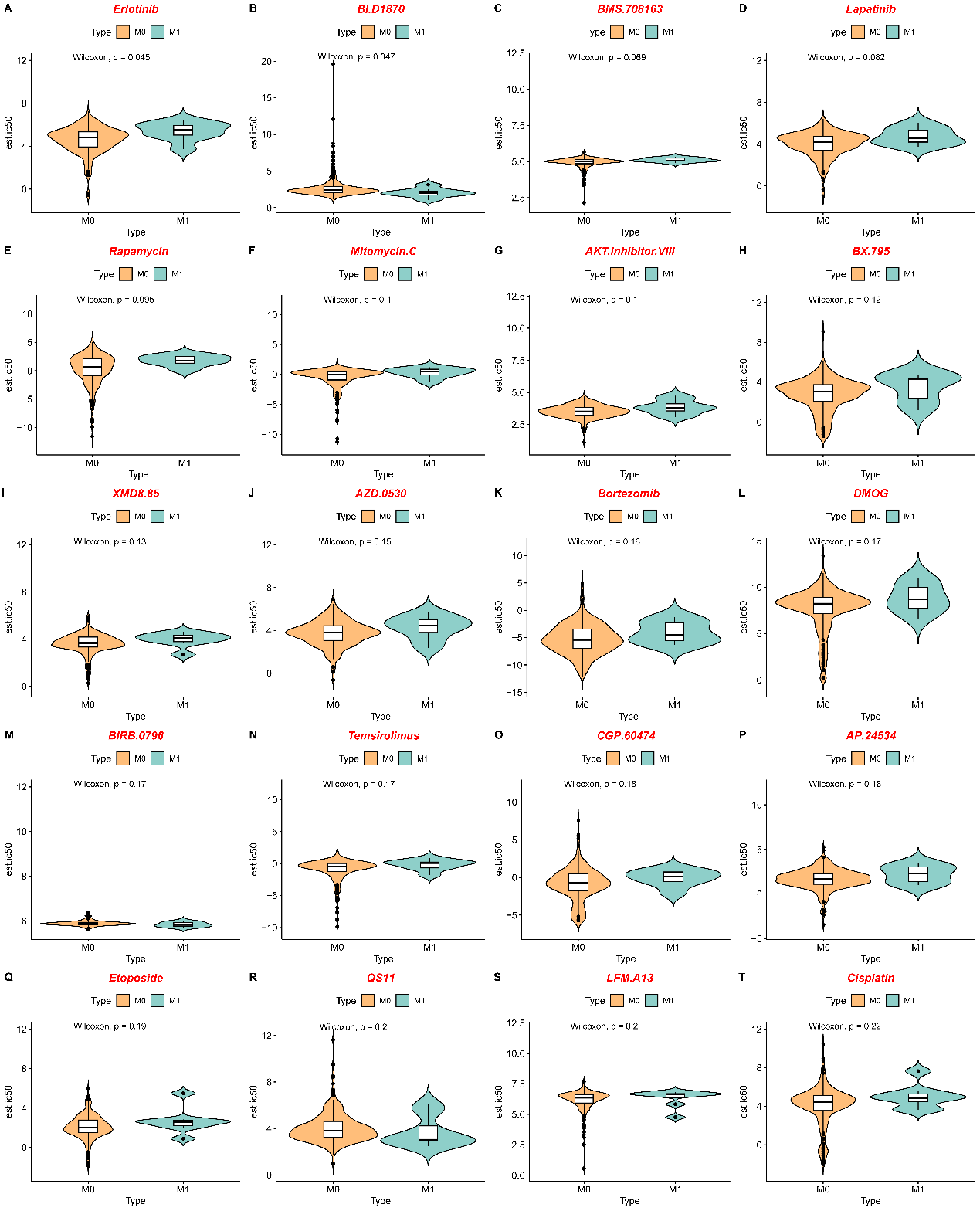


**Figure S1. Drug Sensitivity Analysis of M0 and M1.** Group comparison plots of the sensitivity analysis results of drugs Erlotinib (A), BI.D1870 (B), BMS.708163 ©, Lapatinib (D), Rapamycin (E), Mitomycin.C (F), AKT.inhibitor.VIII (G), BX.795 (H), XMD8.85 (I), AZD.0530 (J), Bortezomib (K), DMOG (L), BIRB.0796 (M), Temsirolimus (N), CGP.60474 (O), AP.24534 (P), Etoposide (Q), QS11 ®, LFM.A13 (S) and Cisplatin (T) for M0 and M1 in disease samples from the TCGA-THCA dataset based on the GDSC database. THCA, Thyroid Cancer; TCGA, The Cancer Genome Atlas. ***p value < 0.001, which is highly statistically significant. Yellow represents M0, green represents M1.
