# Supplementary material for: Estrogen-related genes for thyroid cancer prognosis, immune infiltration, staging, and drug sensitivity
Source: BMC Cancer. 2023 Oct 31;23:1048. doi: 10.1186/s12885-023-11556-0 (PMC10619281; doi:10.1186/s12885-023-11556-0)
Supplement: Supplementary file 6 — Additional file 6: Figure S2. Drug Sensitivity Analysis of N0 and N1. Group comparison plots of the sensitivity analysis results of drugs MK.2206 (A), AZD8055 (B), BIBW2992 ©, X17.AAG (D), PLX4720 (E), Vorinostat (F), Sorafenib (G), AZD6244 (H), ABT.888 (I), AG.014699 (J), JNK.Inhibitor.VIII (K), Nutlin.3a (L), GDC0941 (M), Metformin (N), SL.0101.1 (O), Thapsigargin (P), IPA.3 (Q), CHIR.99021 ®, WO2009093972 (S) and KU.55933 (T) for N0 and N1 in disease samples from the TCGA-THCA dataset based on the GDSC database. THCA, Thyroid Cancer; TCGA, The Cancer Genome Atlas. ***p value < 0.001, which is highly statistically significant. Yellow represents N0, green represents N1. [file 12885_2023_11556_MOESM6_ESM.docx]

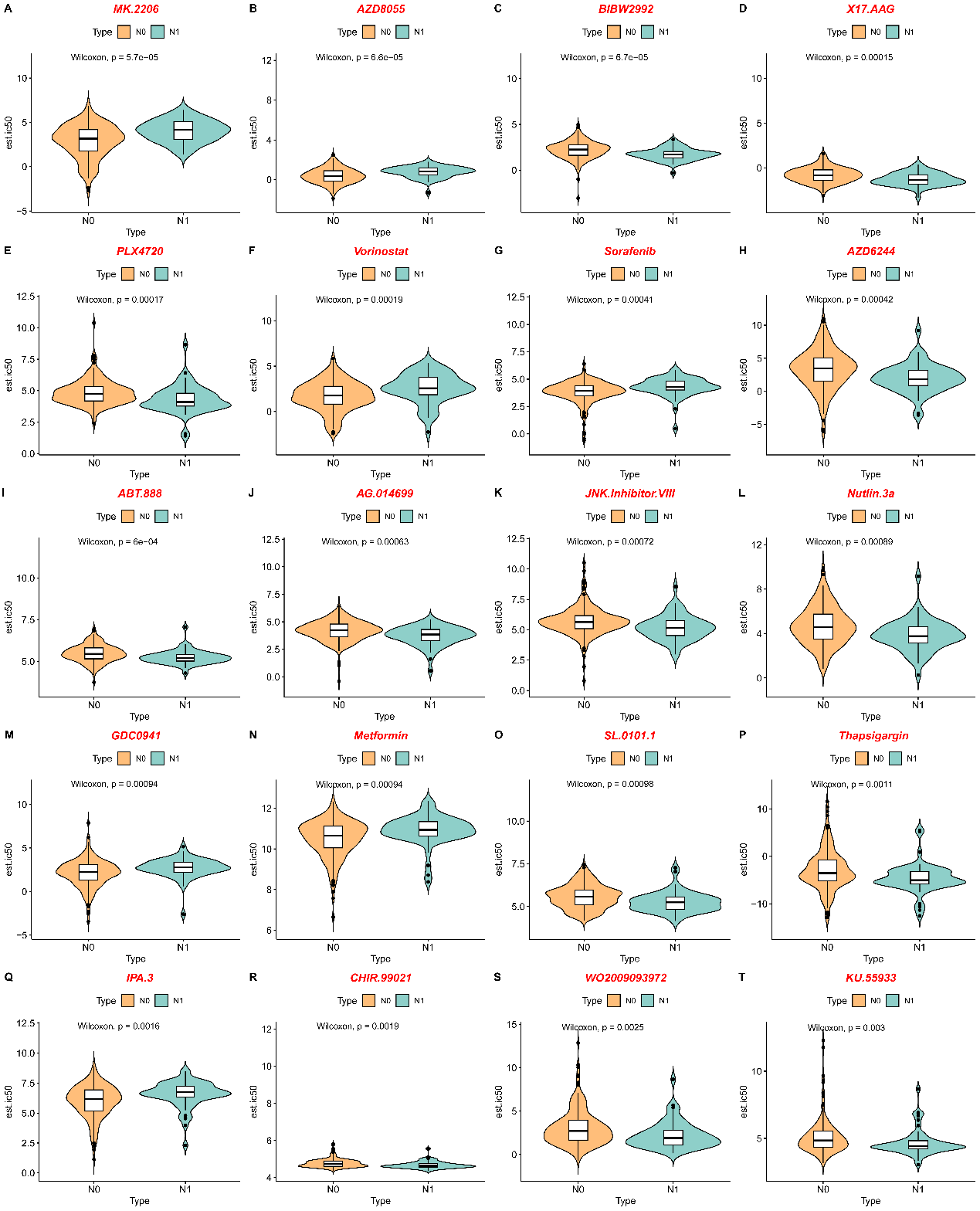


**Figure S2. Drug Sensitivity Analysis of N0 and N1.** Group comparison plots of the sensitivity analysis results of drugs MK.2206 (A), AZD8055 (B), BIBW2992 ©, X17.AAG (D), PLX4720 (E), Vorinostat (F), Sorafenib (G), AZD6244 (H), ABT.888 (I), AG.014699 (J), JNK.Inhibitor.VIII (K), Nutlin.3a (L), GDC0941 (M), Metformin (N), SL.0101.1 (O), Thapsigargin (P), IPA.3 (Q), CHIR.99021 ®, WO2009093972 (S) and KU.55933 (T) for N0 and N1 in disease samples from the TCGA-THCA dataset based on the GDSC database. THCA, Thyroid Cancer; TCGA, The Cancer Genome Atlas. ***p value < 0.001, which is highly statistically significant. Yellow represents N0, green represents N1.
