# Supplementary material for: Estrogen-related genes for thyroid cancer prognosis, immune infiltration, staging, and drug sensitivity
Source: BMC Cancer. 2023 Oct 31;23:1048. doi: 10.1186/s12885-023-11556-0 (PMC10619281; doi:10.1186/s12885-023-11556-0)
Supplement: Supplementary file 7 — Additional file 7: Figure S3. Drug Sensitivity Analysis of Stage I Stage II Stage III and Stage IV. Group comparison plots of the sensitivity analysis results of drugs KU.55933 (A), Etoposide (B), BIBW2992 (C), PF.562271 (D), PD.0332991 (E), Bosutinib (F), Erlotinib (G), Doxorubicin (H), Bleomycin (I), NU.7441 (J), Dasatinib (K), AMG.706 (L), Roscovitine (M), BI.2536 (N), Tipifarnib (O), A.443654 (P), AZD6244 (Q), CI.1040 (R), Gemcitabine (S) and CHIR.99021 (T) for Stage I, Stage II, Stage III and Stage IV in disease samples from the TCGA-THCA dataset based on the GDSC database. THCA, Thyroid Cancer; TCGA, The Cancer Genome Atlas. *** indicates p value < 0.001, which is highly statistically significant. Yellow represents Stage I, blue represents Stage II, purple represents Stage III, green represents Stage IV. [file 12885_2023_11556_MOESM7_ESM.docx]

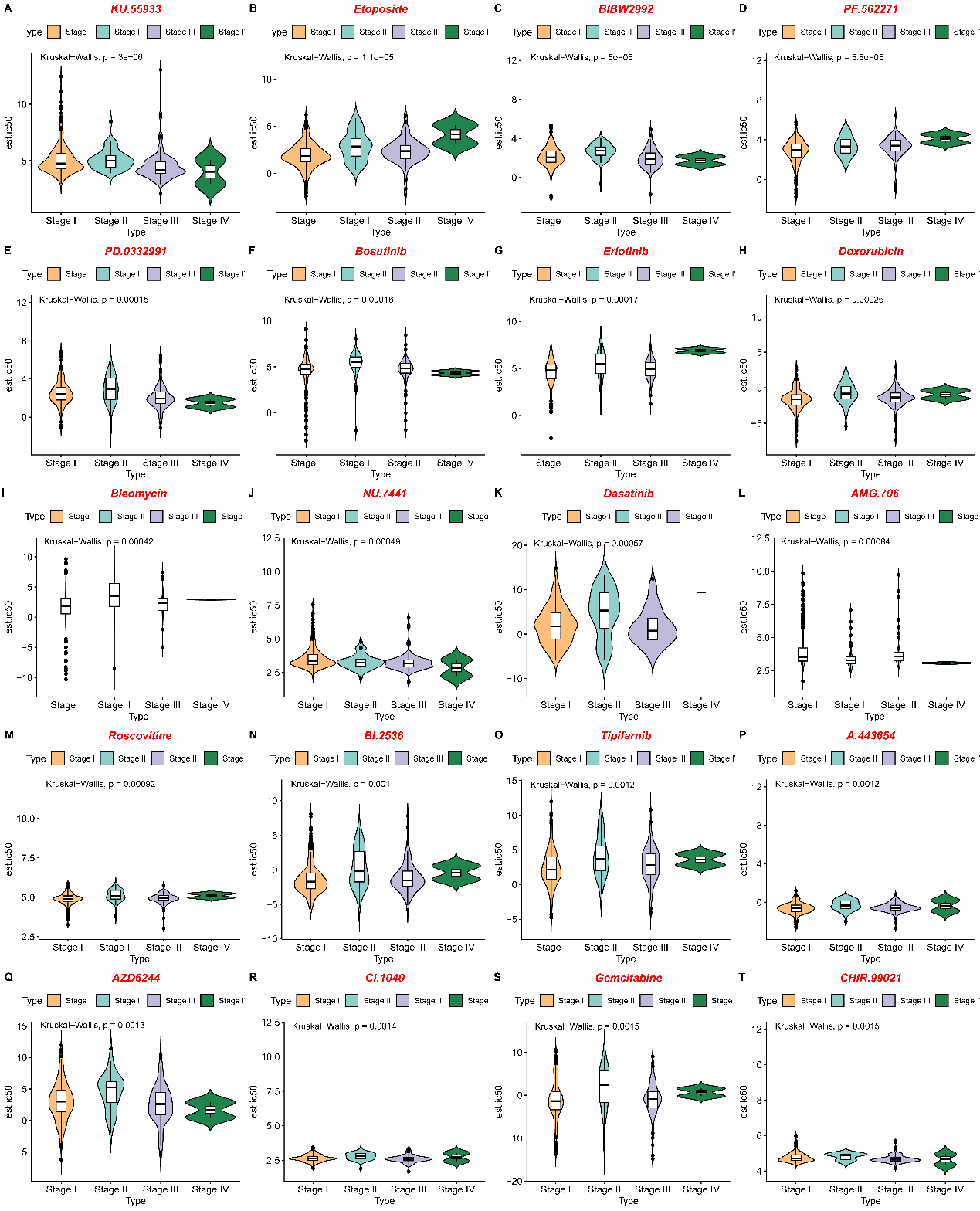


**Figure S4. Drug Sensitivity Analysis of Stage I Stage II Stage III and Stage IV.** Group comparison plots of the sensitivity analysis results of drugs KU.55933 (A), Etoposide (B), BIBW2992 (C), PF.562271 (D), PD.0332991 (E), Bosutinib (F), Erlotinib (G), Doxorubicin (H), Bleomycin (I), NU.7441 (J), Dasatinib (K), AMG.706 (L), Roscovitine (M), BI.2536 (N), Tipifarnib (O), A.443654 (P), AZD6244 (Q), CI.1040 (R), Gemcitabine (S) and CHIR.99021 (T) for Stage I, Stage II, Stage III and Stage IV in disease samples from the TCGA-THCA dataset based on the GDSC database. THCA, Thyroid Cancer; TCGA, The Cancer Genome Atlas. *** indicates p value < 0.001, which is highly statistically significant. Yellow represents Stage I, blue represents Stage II, purple represents Stage III, green represents Stage IV.
