# Supplementary material for: Estrogen-related genes for thyroid cancer prognosis, immune infiltration, staging, and drug sensitivity
Source: BMC Cancer. 2023 Oct 31;23:1048. doi: 10.1186/s12885-023-11556-0 (PMC10619281; doi:10.1186/s12885-023-11556-0)
Supplement: Supplementary file 8 — Additional file 8: Figure S4. Drug Sensitivity Analysis of T1 T2 T3 and T4. Group comparison plots of the sensitivity analysis results of drugs KU.55933 (A), MK.2206 (B), Vorinostat (C), JNK. Inhibitor. VIII (D), Metformin (E), IPA.3 (F), PLX4720 (G), SL.0101.1 (H), SB.216763 (I), NU.7441 (J), Rapamycin (K), Sorafenib (L), WO2009093972 (M), GSK269962A (N), JNJ.26854165 (O), ABT.888 (P), AZD6244 (Q), ATRA (R), PF.02341066 (S) and Elesclomol (T) for T1, T2, T3 and T4 in disease samples from the TCGA-THCA dataset based on the GDSC database. THCA, Thyroid Cancer; TCGA, The Cancer Genome Atlas. *** indicates p value < 0.001, which is highly statistically significant. Yellow represents T1I, blue represents T2, purple represents T3, green represents T4. [file 12885_2023_11556_MOESM8_ESM.docx]

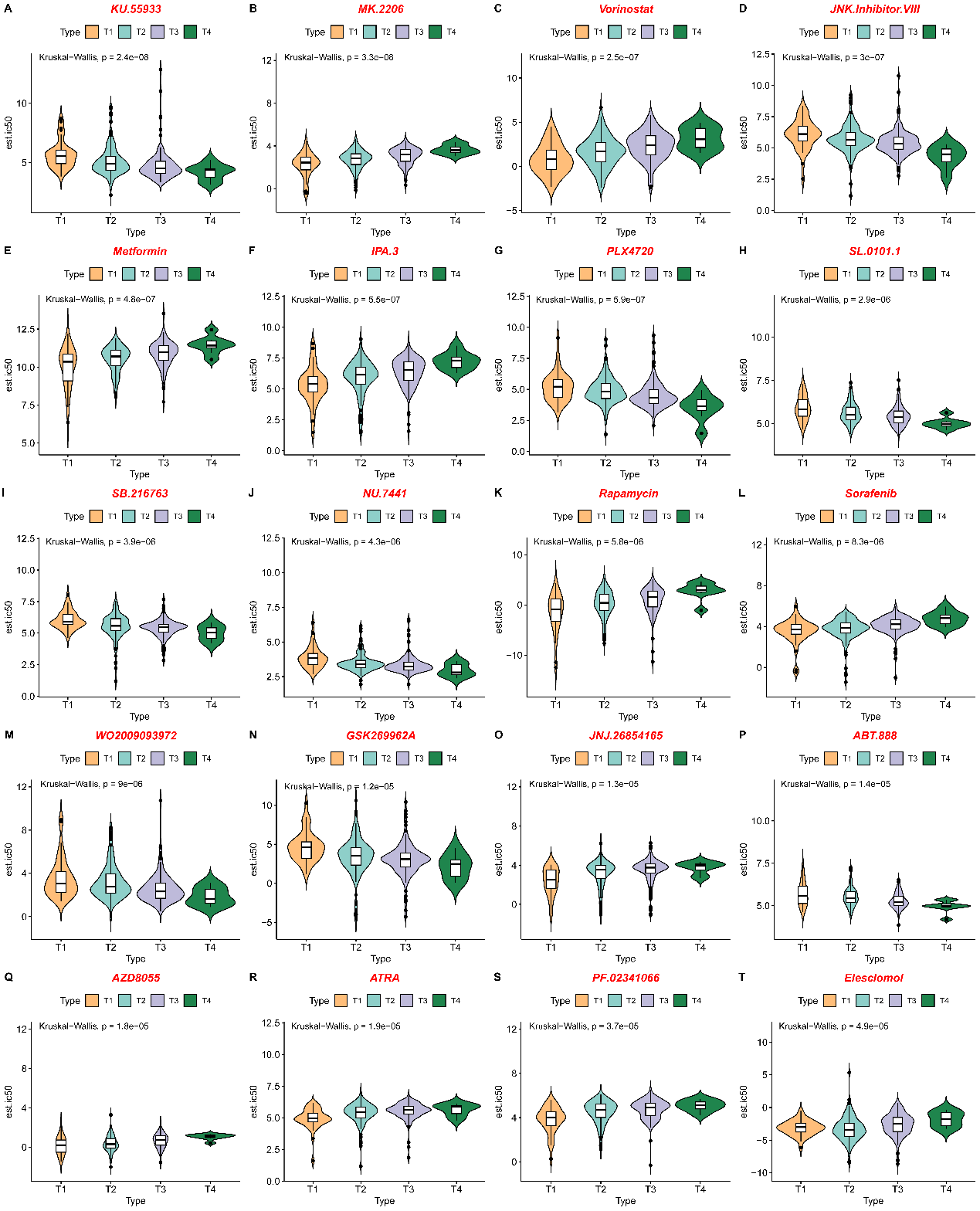


**Figure S4. Drug Sensitivity Analysis of T1 T2 T3 and T4.** Group comparison plots of the sensitivity analysis results of drugs KU.55933 (A), MK.2206 (B), Vorinostat (C), JNK.Inhibitor.VIII (D), Metformin (E), IPA.3 (F), PLX4720 (G), SL.0101.1 (H), SB.216763 (I), NU.7441 (J), Rapamycin (K), Sorafenib (L), WO2009093972 (M), GSK269962A (N), JNJ.26854165 (O), ABT.888 (P), AZD6244 (Q), ATRA (R), PF.02341066 (S) and Elesclomol (T) for T1, T2, T3 and T4 in disease samples from the TCGA-THCA dataset based on the GDSC database. THCA, Thyroid Cancer; TCGA, The Cancer Genome Atlas. *** indicates p value < 0.001, which is highly statistically significant. Yellow represents T1I, blue represents T2, purple represents T3, green represents T4.
